# An IRES-like cis-acting element located within the EV-A71 coding region drives translation independent of the 5′-IRES and modulates viral fitness through regulated binding of viral RNA to 3D polymerase

**DOI:** 10.1128/jvi.00355-26

**Published:** 2026-05-27

**Authors:** Yifan Xing, Qi Liu, Meixian Fu, Hao Zheng, Tong Zhao, Xixi Chen, Xia Cai, Jian-Er Long

**Affiliations:** 1 Key Laboratory of Medical Molecular Virology (MOE/NHC/CAMS), School of Basic Medical Sciences, Shanghai Medical College, Fudan University 12478, Shanghai, China; 2 Department of Medical Microbiology and Parasitology, School of Basic Medical Sciences, Shanghai Medical College, Fudan University 12478, Shanghai, China; 3 Shanghai Institute of Infectious Disease and Biosecurity, Fudan University 12478, Shanghai, China; 4 Biosafety Level-3 Laboratory, Fudan University 12478, Shanghai, China; Emory University School of Medicine 12239, Atlanta, Georgia, USA

**Keywords:** enterovirus A71, IRES-like cis-acting element, IRES-independent translation, RNA structure, 3D polymerase, infectious clone, viral fitness

## Abstract

Enterovirus A71 (EV-A71) is a leading cause of hand, foot, and mouth disease and is occasionally associated with severe neurological complications in young children. Previous studies of EV-A71 and related enteroviruses suggested that the virus possibly contains additional cis-acting elements (CAEs) within the major open reading frame (ORF) regulating viral protein translation. To systematically explore CAEs in the EV-A71 genome and characterize their functions, we utilized bicistronic reporter plasmids to screen the entire genome, identifying internal ribosome entry site (IRES)-like CAEs within the VP3–VP1 and 2A–2B coding regions. We demonstrate that EV-A71 translation can occur independently of the 5′-IRES and that the element within 2A–2B specifically facilitates this alternative initiation. Furthermore, the IRES-like CAE significantly modulates viral propagation by altering the expression of viral proteins including VP1 and 3D polymerase and shifting the ratio of positive-sense to negative-sense genomic RNA. Disruption of its RNA structure via synonymous mutations reduced viral genome replication, progeny production, and fitness. Mechanistically, these impairments correlated with decreased RNA binding to the viral 3D polymerase. These findings illuminate the multifaceted roles of coding-region IRES-like CAEs in viral infection and fitness, reveal a noncanonical mechanism for enteroviral protein synthesis independent of the 5′-IRES, and provide a framework to identify RNA structures and synonymous mutations for attenuated vaccine design and antiviral strategies.

**IMPORTANCE:**

The biological significance of cis-acting elements (CAEs) within viral coding regions, particularly their impact on infectivity and fitness, remains poorly understood. Here, we characterize an internal ribosome entry site (IRES)-like CAE in Enterovirus A71 (EV-A71), the causative agent of hand, foot, and mouth disease. Through genome-wide screening, we identified novel IRES-like CAEs and demonstrate that EV-A71 translation can proceed independently of the 5′-IRES, with the 2A–2B element specifically mediating this alternative initiation. The 2A–2B CAE and its associated RNA secondary structure are essential for viral infectivity and fitness. Synonymous mutations disrupting local stem-loop structures impair viral RNA binding to the 3D polymerase, resulting in reduced fitness. These findings reveal a noncanonical mechanism for IRES-independent enteroviral protein synthesis, elucidate how RNA structures and synonymous mutations in coding-region CAEs modulate viral replication, and inform attenuated vaccine design and antiviral strategies while revealing the evolutionary implications of these regulatory elements.

## INTRODUCTION

In positive-sense RNA (+ssRNA) viruses, cis-acting elements (CAEs) are functional RNA structures or sequences embedded within the viral genome (1). Unlike trans-acting factors—typically diffusible proteins or RNAs that act on multiple targets—CAEs function exclusively in *cis*, exerting their influence only on the same RNA molecule that contains them. These elements enable viruses to overcome fundamental gene expression constraints, such as the absence of a 5′ cap, by facilitating cap-independent translation through mechanisms including internal ribosome entry sites (IRESs) in picornaviruses and flaviviruses and 3′ cap-independent translation enhancers (3′ CITEs) in members of the family *Tombusviridae* (2, 3). CAEs also regulate other stages of the viral life cycle. For instance, cis-acting replication elements (CREs) in picornaviruses are specific RNA structures recognized by the viral RNA-dependent RNA polymerase (RdRp) and other replication proteins to serve as platforms for replication complex assembly and initiation of RNA synthesis (4, 5). Additionally, many RNA viruses, including coronaviruses and retroviruses, employ programmed ribosomal frameshifting (PRF) to regulate the expression of multiple proteins from a single mRNA. This process is directed by a frameshift signal comprising a “slippery” sequence and downstream RNA secondary structure that induces ribosomal pausing (6–8).

These CAEs range from simple primary sequences to complex secondary and tertiary structures, including stem-loops, pseudoknots, and riboswitches. Such structures mediate specific interactions with viral proteins, host factors, and other RNA molecules, thereby regulating nearly every stage of the viral life cycle, including gene expression, genome replication, and encapsidation (1, 9). Consequently, considerable efforts have been devoted to identifying potential CAEs within viral genomes using high-throughput sequencing combined with structural modeling, as well as bicistronic reporter assays to assess whether candidate sequences drive downstream translation. For example, Weingarten-Gabbay et al. (10) developed a high-throughput bicistronic assay to quantify cap-independent translation, demonstrating that IRES-like CAEs are widely distributed across coding regions of +ssRNA viruses, including picornaviruses. They proposed that these viruses can initiate translation internally to generate partial proteins in addition to the canonical full-length polyprotein. Similarly, an IRES has been proposed within the VP3 coding region of human rhinovirus 16 (HRV16), a related picornavirus, that directs reporter gene expression *in vitro*; however, whether this element mediates authentic viral translation remains unresolved (11). These observations prompted us to hypothesize that the enterovirus genome encodes additional IRES-like CAEs within its coding region. We tested this using EV-A71 as a model system.

Enterovirus A71 (EV-A71) is a positive-sense RNA virus belonging to the species Enterovirus A within the family *Picornaviridae*. EV-A71 infection typically causes hand, foot, and mouth disease in children, with severe cases manifesting neurological complications including meningitis, encephalitis, acute flaccid paralysis, and pulmonary edema (12, 13). The virus has circulated globally, with particularly high prevalence in the Asia-Pacific region over the past decades (14). Given the absence of approved antivirals (15) and the limited efficacy of licensed vaccines in China against specific genotypes (16), understanding EV-A71 genome organization and infectivity is crucial for advancing the knowledge of viral replication and developing control strategies. The EV-A71 genome contains a small upstream open reading frame (uORF) partially overlapping upstream of a large open reading frame (ORF) flanked by 5′ and 3′ untranslated regions (UTRs) (17, 18), This major ORF encodes a polyprotein precursor that undergoes proteolytic processing to generate structural proteins (P1 region; cleaved to VP4, VP2, VP3, and VP1) and nonstructural proteins (P2 and P3 regions; processed to 2A–2C and 3A–3D, respectively). The P1–P2 junction is cleaved primarily by the 2A protease, whereas 3C protease (or its precursor 3CD) mediates cleavage at other sites. The 3D protein functions as the viral RNA-dependent RNA polymerase (RdRp), which is essential for genome replication (14).

In this study, we screened the EV-A71 coding region for potential CAEs using a bicistronic reporter assay and characterized a novel IRES-like element. We demonstrate that EV-A71 translation can occur independently of the canonical 5′ IRES and that the element within 2A–2B specifically facilitates this alternative initiation. Beyond functioning as a weak IRES, this CAE significantly modulates viral infectivity and fitness, as demonstrated by synonymous mutations that disrupt its RNA stem-loop structure. These phenotypic effects correlated with reduced binding affinity between viral RNA and the 3D polymerase.

## RESULTS

### Identification of novel cis-acting elements in the coding region of THE EV-A71 genome

**Fig 1:**
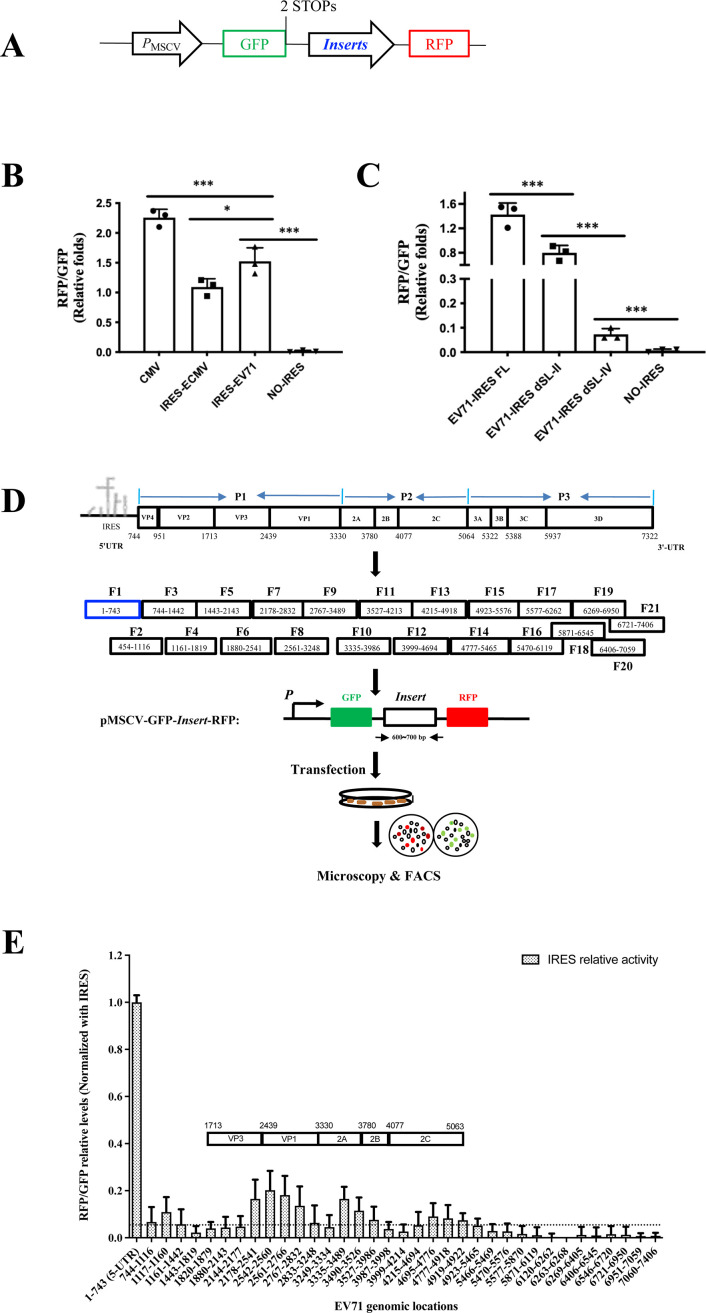
Identification of IRES-like cis-acting elements in the EV-A71 genome. (**A**) Schematic of the bicistronic reporter plasmid pMSCV-*GFP*-Insert-*RFP*. (**B**) Regulatory activity of indicated cis-acting elements, expressed as the ratio of RFP- to GFP-positive cells. Data represent the mean ± SD from three independent experiments. (**C**) Effect of IRES deletions on regulatory activity. Wild-type EV-A71 5′-UTR (nt 1–743) or mutants lacking stem-loop II (Δnt 129–167) or stem-loop IV (Δnt 445–561) were inserted into the reporter. Plasmids (3 µg) were transfected into 293FT cells in 6-well plates; RFP expression was quantified at 72 hours post-transfection (hpt). ΔIRES indicates the empty vector control. (**D**) Schematic of the genome scanning strategy. The EV-A71 coding region was tiled with 21 overlapping fragments (F1–F21); the 5′ UTR served as a positive control. (**E**) Distribution of regulatory activity across the EV-A71 genome. Activity is expressed as the percentage of the 5′-UTR positive control (set to 100%) and mapped to nucleotide position. Data were averaged across the length of each fragment.

High-throughput screening has revealed IRES-like elements within coding regions of RNA viruses, including members of the *Picornaviridae* family (10). We hypothesized that EV-A71 similarly encodes IRES-like CAEs within its coding region that modulate viral infection and propagation. To test this, we developed a bicistronic reporter system analogous to that described by Weingarten-Gabbay (10). The construct contained green fluorescent protein (GFP) driven by the murine stem cell virus (MSCV) promoter as an internal control, followed by an intervening sequence (where test elements were inserted) and downstream mCherry red fluorescent protein (RFP) as the reporter (Fig. 1A). Insertion of the CMV promoter or established IRES elements from EMCV or EV-A71 directed RFP expression, whereas empty vector yielded no RFP-positive cells, confirming system specificity (Fig. 1B; Fig. S1A). To validate the correlation between RFP fluorescence and regulatory activity, we generated deletion mutants of the canonical EV-A71 IRES. The EV-A71 IRES comprises six stem-loops (SL II–SL VI) essential for translation initiation: SL IV binds the scaffold protein eIF4G (19), while SL II recruits host factors including hnRNP A1, AUF1, HuR, and Ago to modulate protein expression (20, 21). Deletion of SL II reduced RFP levels compared to wild-type IRES but retained minor activity, whereas SL IV deletion nearly abolished RFP expression (Fig. 1C; Fig. S1B). The results were consistent with previous reports, suggesting that RFP levels are a reliable indicator of the regulatory activity of inserted CAEs.

We screened the EV-A71 genome using this dual-fluorescence system, cloning 21 overlapping cDNA fragments (~600–700 bp) spanning the coding region with ~200 bp overlaps (reflecting the approximate size of the viral IRES) (Fig. 1D). Quantification of GFP and RFP expression following transfection revealed that, in addition to the 5′-IRES, several coding-region fragments exhibited IRES-like activity, albeit at only 10%–20% of the full-length IRES activity (Fig. 1E; Fig. S2A through C). These potential CAEs were nonrandomly distributed, clustering within the VP3–VP1 and 2A–2B regions (Fig. 1E).

### Characterization of IRES-like CAEs and mapping of the primary regulatory domain

We characterized the CAEs within the VP3–VP1 (nt 2178–2832) and 2A–2B (nt 3335–3986) regions. Following transfection, quantitative RT-PCR confirmed the equivalent RNA levels of GFP and RFP (Fig. S3A), and conventional RT-PCR verified that the CAE-containing transcripts maintained expected sizes (Fig. S3B). This indicated that the inserted CAEs were transcribed as a single transcript with the GFP and RFP genes, suggesting the absence of RNA splicing sites within the transcripts; otherwise, we would expect to observe different transcript sizes. To exclude ribosomal read-through from the upstream cistron, we inserted a stem-loop structure known to block scanning ribosomes (22) downstream of the EMCV IRES (Fig. S3C) or upstream of the candidate CAEs (Fig. S3D). This hairpin abolished EMCV IRES-directed luciferase expression but did not impair CAE-directed RFP expression (Fig. S3C and D), confirming that the screened elements function as genuine IRES-like elements rather than promoting cryptic promoter or splicing activity.

**Fig 2:**
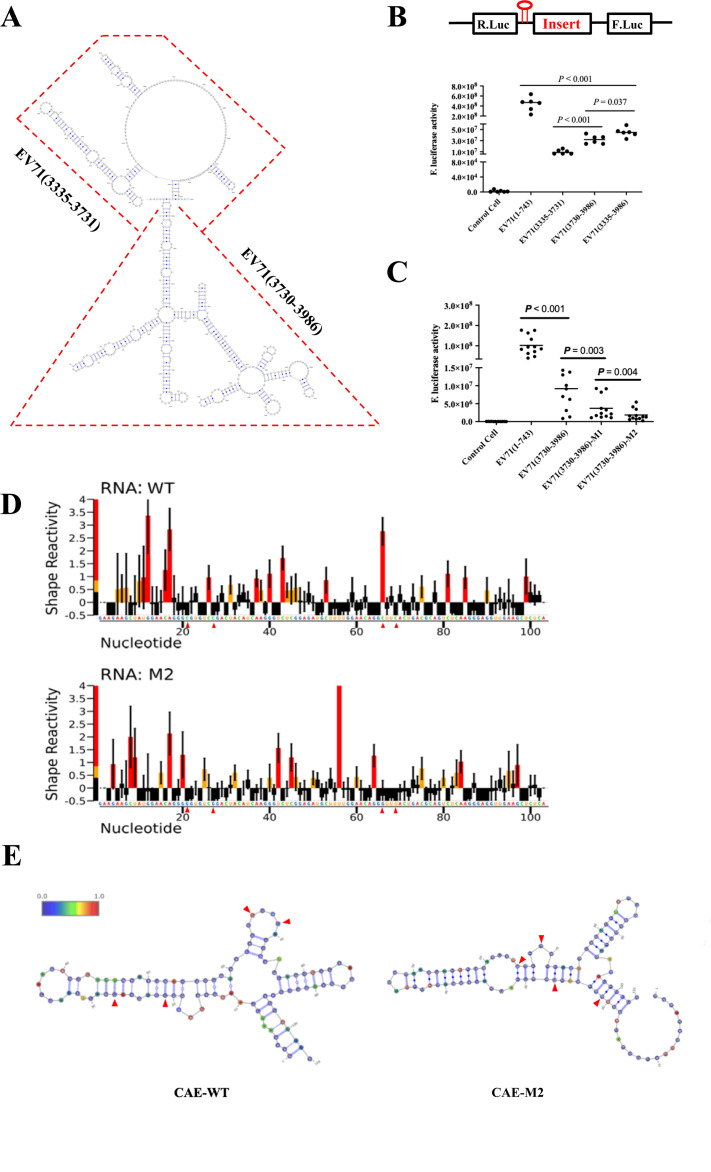
Identification of the primary regulatory domain within the IRES-like cis-acting element. (**A**) Predicted secondary structure of the CAE (nt 3335–3986) analyzed using RNAfold and Vienna RNA, showing Domain I (nt 3335–3731) and Domain II (nt 3730–3986). (**B**) Luciferase reporter assay identifying the primary regulatory domain. Domains I and II were cloned into pMSCV-*Rluc*-Insert-*Fluc* and transfected into 293FT cells. Firefly luciferase activity (normalized to *Renilla*) was measured at 48 hpt. Data represent mean ± SD from two independent experiments performed in triplicate. *P < 0.05 (Student’s *t*-test). (**C**) Effect of synonymous mutations on CAE activity. Luciferase reporters containing wild-type Domain II or M1/M2 mutants were transfected into 293FT cells. Data represent mean ± SD from four independent experiments performed in triplicate. *P < 0.05. (**D**) SHAPE reactivity profiles of wild-type and M2 mutant Domain II RNA, analyzed using ShapeMapper 2. Red triangles indicate mutation sites. (**E**) RNA secondary structure models of wild-type and M2 mutant Domain II generated using RNAfold (v2.4.17).

Given the importance of the 2A–2B region within the nonstructure protein coding region in regulating viral replication, we performed a detailed analysis on this CAE. Bioinformatic analysis by RNAfold and Vienna RNA identified two structural domains: Domain I (nt 3335–3731) and Domain II (nt 3730–3986) (Fig. 2A). To map the functional domain, we cloned each region into a bicistronic *Renilla*–firefly luciferase reporter (pMSCV-*Rluc*-Insert-*Fluc*). While Domain I exhibited minor activity, Domain II (nt 3730–3986) retained the major capacity of the complete CAE, identifying it as the primary functional domain (Fig. 2B). We next introduced synonymous mutations to disrupt Domain II structure. Among 20 combinatorial mutants tested, two (M1 and M2) significantly altered predicted secondary structures. Both contained four synonymous substitutions within the 2B coding sequence: M1 (C3782T, C3788T, C3827T, and C3830T) and M2 (C3782G, C3788G, C3827G, and C3830T). These mutations reduced the number of stem-loop arms in the 3′-subdomain from five (wild-type) to three (M1) or four (M2) (Fig. 2A; Fig. S4A and B). Notably, while these individual substitutions represent natural polymorphisms observed in clinical isolates, the complete M2 mutation set is not commonly detected in such isolates (Fig. S4C). Functional analysis revealed that structural disruption correlated with reduced luciferase activity, with M2 exhibiting more severe impairment than M1 (Fig. 2C; Fig. S4D). We validated these structural alterations using selective 2′-hydroxyl acylation analyzed by primer extension (SHAPE) (23, 24). This technique employs electrophilic reagents to modify conformationally flexible nucleotides, with adduct locations detected as reverse transcription termination sites (25). SHAPE profiles of Domain II revealed significant differences between wild-type and M2 mutant RNAs proximal to the mutation cluster, especially within 15 bp upstream of the third mutation (Fig. 2D), confirming substantial remodeling of stem lengths, stem number, and loop geometries (Fig. 2E).

### EV-A71 3D translation proceeds when 5′-IRES-mediated translation is blocked by lethal mutations

**Fig 3:**
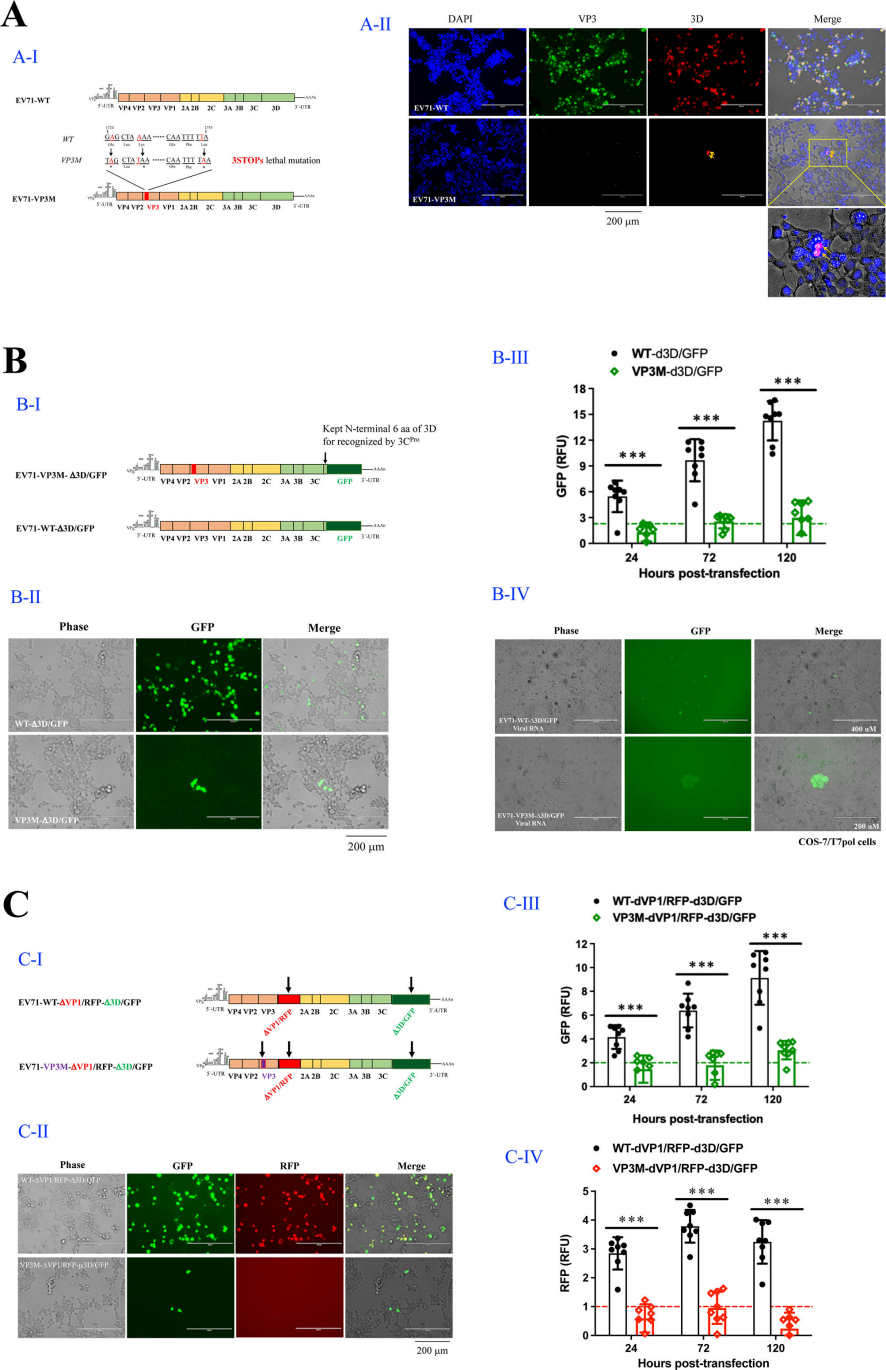
IRES-independent translation of the EV-A71 3D gene locus. (**A**) I. Schematic of the infectious clone EV-A71-WT and the VP3-lethal mutant (EV-A71-VP3M) containing three premature stop codons in VP3. II. Immunofluorescence staining for VP3 (green) and 3D (red) in 293FT/T7pol cells transfected with the indicated clones (3 µg/well, 6-well plate) and fixed at 16 hpt. Insets show magnified views; arrows indicate 3D-positive cells. (**B**) I. Schematic of EV-A71-WT-Δ3D/GFP and EV-A71-VP3M-Δ3D/GFP, in which the 3D coding sequence was replaced with *GFP*. II. GFP expression at 24 hpt. III. Quantification of GFP fluorescence (relative fluorescence units, RFU) at indicated time points. Data represent mean ± SD from two independent experiments performed in quadruplicate. The dashed line indicates the threshold (3× SD above mock transfection). ***P < 0.001 (Student’s *t*-test). IV. GFP expression in COS-7/T7pol cells transfected with *in vitro*-transcribed RNA from the indicated clones, imaged at 12 hpt. (**C**) I. Schematic of EV-A71-ΔVP1/RFP-Δ3D/GFP derivatives. The *VP1* was replaced with *RFP* in the background of the clones described in panel B-I. II. GFP and RFP expression at 24 hpt. III. Quantification of fluorescence as in panel B-III. Thresholds were set at 3× SD (GFP) or 2.1× SD (RFP) above mock. ***P < 0.001.

Canonical enteroviral polyprotein translation is directed by the 5′-UTR IRES (4). However, previous studies suggested that EV-A71 may utilize IRES-independent mechanisms (26), though direct evidence for *de novo* viral protein synthesis via these alternative pathways was lacking. To determine whether IRES-like CAEs can initiate translation independently of the 5′-IRES, we developed a T7 polymerase-dependent reverse genetics system utilizing two components: (i) a pcDNA3.1-based EV-A71 infectious clone in which the CMV promoter was replaced with a T7 promoter and (ii) 293FT cells stably expressing T7 polymerase (293FT/T7pol). This system enabled efficient virus rescue (titers up to 9.8 × 10⁹ PFU/mL) following single-plasmid transfection (27). To test whether IRES-like CAEs within the VP3–VP1 and 2A–2B regions could drive downstream gene expression, we blocked 5′-IRES-mediated translation using lethal mutations in VP3 (EV71-VP3M), introducing three stop codons at the N terminus of VP3 (Fig. 3A, panel I). As expected, VP3 was undetectable by immunostaining in EV71-VP3M-transfected cells. Strikingly, however, 3D expression was still observed, albeit at a much lower efficiency than in the wild-type strain (Fig. 3A, panel II). To facilitate the detection of 3D expression without confounding by viral replication, we generated a translation reporter system in which the 3D coding sequence was replaced with *GFP* (WT-d3D/GFP) and introduced the VP3 lethal mutation into this background (VP3M-d3D/GFP) (Fig. 3B, panel I). GFP expression was readily detected in WT-d3D-/GFP-transfected cells and at markedly reduced levels in VP3M-d3D-/GFP-transfected cells (Fig. 3B, panel II). GFP fluorescence marginally increased over time post-transfection (Fig. 3B, panel III). GFP expression was also observed—at even lower efficiency—in COS-7 cells transfected with *in vitro-*transcribed RNA from VP3M-d3D/GFP and WT-d3D/GFP templates (Fig. 3B, panel IV), confirming that expression occurs in the absence of plasmid DNA- and 5′-IRES-mediated translation.

To distinguish which IRES-like CAE (VP3–VP1 versus 2A–2B) mediated this residual translation, we replaced the VP1 coding sequence with *RFP* in the VP3M-d3D/GFP background (VP3M-dVP1/RFP-d3D/GFP) (Fig. 3C, panel I). In cells transfected with this construct, we observed GFP expression (substituting for 3D), but not RFP (substituting for VP1) (Fig. 3C, panel II); conversely, both fluorophores were co-expressed in the wild-type background. GFP expression persisted and marginally increased over time (Fig. 3C, panel III), whereas RFP remained undetectable in VP3M-dVP1-/RFP-d3D-/GFP-transfected cells (Fig. 3C, panel IV). These results indicate that the IRES-like CAE within the 2A–2B region, rather than that within VP3–VP1, drives residual 3D translation following blockade of 5′-IRES-mediated read-through.

### The IRES-like CAE facilitates 3D translation independently of 5′-IRES

**Fig 4:**
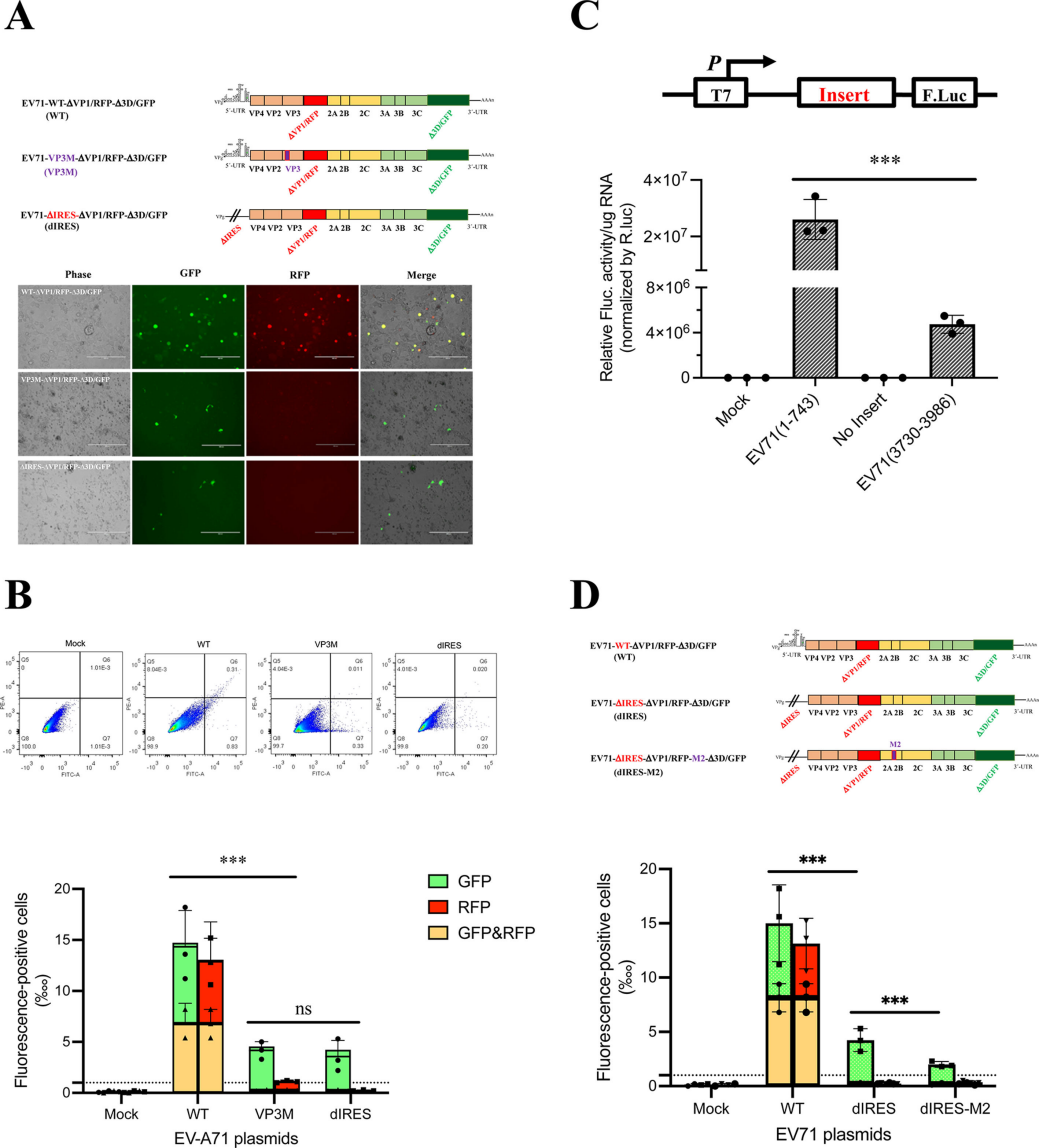
EV-A71 translation occurs independently of the canonical IRES and is directed by the IRES-like CAE within the 2A–2B region. (**A**) Schematic (upper panel) of IRES-deleted (dIRES) and VP3-lethal mutant (VP3M) derivatives in which *VP1* was replaced with *RFP* and *3D* was replaced with *GFP*. Lower panel: GFP and RFP expression in COS-7/T7pol cells transfected with the indicated clones (4 μg/well, 6-well plate) at 56 hpt. (**B**) Quantification of GFP and RFP by flow cytometry following transfection as in panel A. The upper panel shows a representative of three independent experiments. ***P < 0.001 (Student’s *t*-test). (**C**) Cell-free translation directed by the 2A–2B CAE. The CAE, EV-A71 5′-UTR (positive control), or empty vector (negative control) was cloned upstream of the firefly luciferase coding sequence in pcDNA3.1. Linearized plasmids were transcribed *in vitro* and translated in the rabbit reticulocyte lysate. (**D**) Upper panel: Schematic of the mutant dIRES-M2 derived from dIRES. Lower panel: GFP and RFP expression quantified by flow cytometry following transfection as in panel A. ***P < 0.001.

While GFP expression from the 3D locus was detected following the blockade of 5′-IRES-mediated translation, we could not exclude RNA circularization mediated by the 5′-UTR IRES as the initiating mechanism (28). To distinguish these possibilities, we generated IRES-deletion variants (dIRES) of the EV71-WT-dVP1/RFP-d3D/GFP reporter clone (Fig. 4A, upper panel). Following transfection into COS-7 cells stably expressing T7 polymerase (COS-7/T7pol), both RFP and GFP were robustly co-expressed in the wild-type IRES-containing construct. By contrast, only GFP was observed—at markedly reduced levels—in VP3M and dIRES constructs, whereas RFP expression from the VP1 locus was undetectable (Fig. 4A, lower panel). Flow cytometric analysis confirmed GFP expression occurred independently of 5′-IRES, albeit at substantially reduced levels compared to the intact IRES construct (Fig. 4B). These results demonstrate that functional IRES-like CAEs are located downstream of VP1; given that VP1 was replaced with RFP in this system, the 2A–2B region likely drives residual 3D translation. Notably, GFP expression was barely detectable when IRES-deleted clones were transfected into 293FT/T7pol cells (data not shown), indicating that IRES-independent translation is restricted by unknown host factors in a cell-type-specific manner.

As shown in Fig. 2C, Domain II of the 2A–2B CAE directed downstream reporter expression *in vivo*. To determine whether this activity was RNA-intrinsic, we performed *in vitro* transcription-translation assays. Reporter constructs containing the 2A–2B CAE, the EV71 IRES, or no insert upstream of firefly luciferase were transcribed by T7 polymerase and translated in the rabbit reticulocyte lysate. The 2A–2B CAE initiated firefly luciferase expression, but at only ~18% of the activity observed with the EV71 IRES (Fig. 4C), consistent with results from our bicistronic screening assays (Fig. 1E). To test whether the 2A–2B CAE specifically mediates 3D locus translation, we introduced the M2 synonymous mutations into the dIRES background (dIRES-M2) and quantified fluorescence by flow cytometry. The M2 mutation significantly reduced GFP-positive cells under IRES-independent conditions (Fig. 4D), indicating that the 2A–2B IRES-like CAE plays a critical role in driving 3D translation via a structure-dependent mechanism.

### Structural perturbation of the CAE impairs viral production by dysregulating genome replication and protein expression

**Fig 5:**
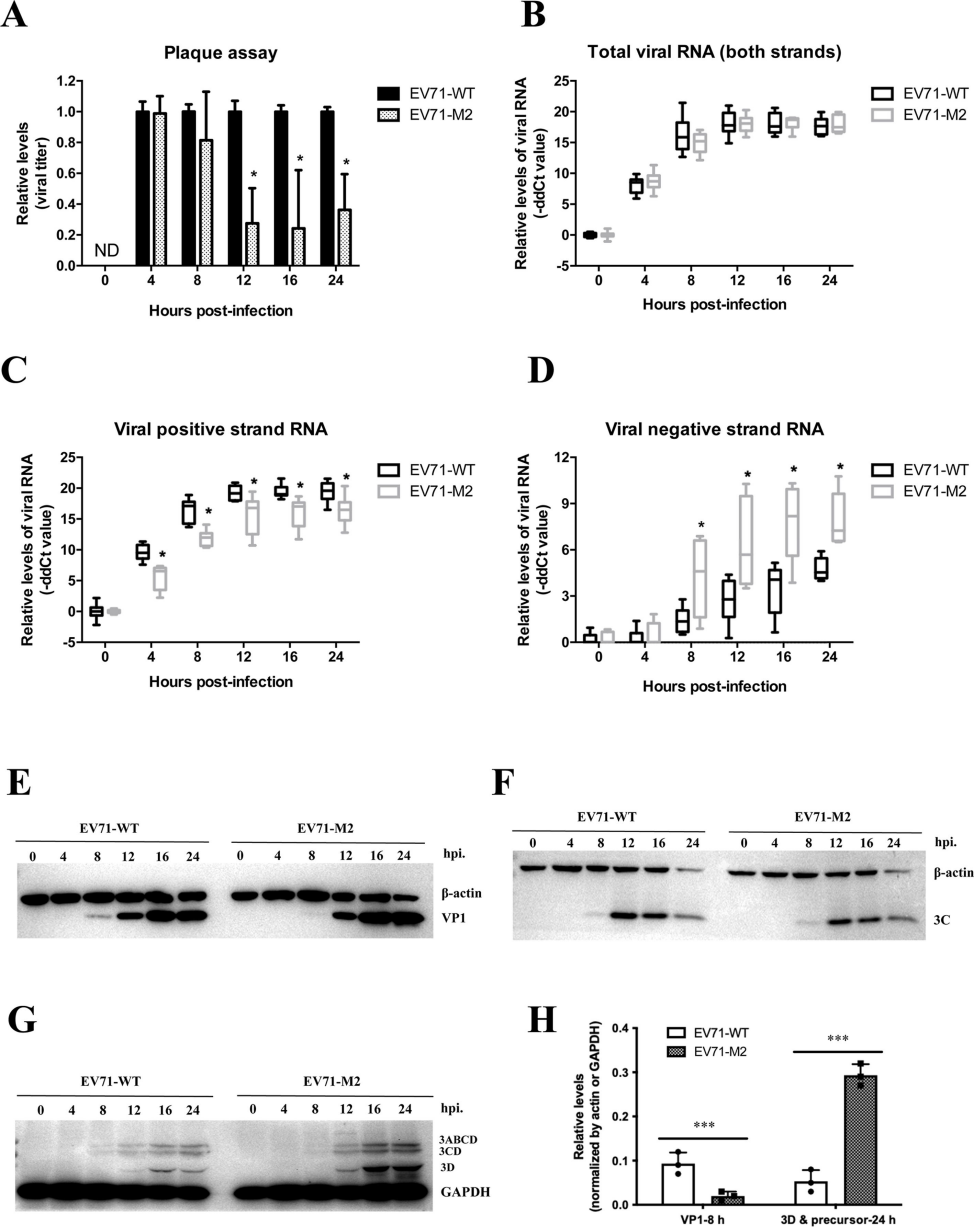
Impact of the EV-A71-M2 mutation on viral titer, genome replication, and protein expression in RD cells. (**A**) Viral titers in culture supernatants determined by plaque assay. RD cells were infected at a multiplicity of infection (MOI) of 20 PFU/cell, and supernatants were harvested at the indicated time points. (**B**) Total viral RNA quantified by qPCR. (**C and D**) Positive-sense (**C**) and negative-sense (**D**) viral RNA quantified by strand-specific qPCR. (**E–G**) Immunoblot detection of VP1 (**E**), 3C (**F**), and 3D (**G**). β-actin or GAPDH served as loading controls. (**H**) Quantification of VP1 and 3D protein levels from panels E and G. Data represent mean ± SD from three independent experiments. *P < 0.05; ***P < 0.001 (Student’s *t*-test).

The M2 synonymous mutations disrupt CAE RNA structure and impair downstream gene expression (Fig. 2C). To determine whether these structural alterations affect viral replication, we rescued EV-A71-M2 (containing the M2 mutations) alongside wild-type EV-A71 (EV-A71-WT) (27) and infected RD cells. Plaque assays revealed that EV-A71-M2 produced 3- to 5-fold lower titers than wild-type at 12–24 hours post-infection (hpi) (Fig. 5A), despite unchanged total viral RNA levels (Fig. 5B). Strand-specific quantitative PCR showed a significant decrease in positive-sense genomic RNA and a concomitant increase in negative-sense antigenomic RNA in the mutant (Fig. 5C and D), suggesting dysregulated replication complex function. Analysis of viral protein expression revealed differential effects across the polyprotein. VP1, located upstream of the 2A–2B CAE, accumulated earlier in wild-type-infected cells than in EV-A71-M2-infected cells (Fig. 5E through H). Levels of 3C protease (downstream of the CAE) were modestly reduced in the mutant (Fig. 5F). Paradoxically, 3D polymerase accumulated to substantially higher levels in EV-A71-M2-infected cells at later time points (Fig. 5G and H), indicating that CAE disruption alters polymerase expression dynamics or turnover.

To determine whether the effects of M2 mutation was cell-type-specific, we repeated these experiments in human neuroblastoma SH-SY5Y cells. Consistent with RD cell data, EV-A71-M2 exhibited modestly reduced titers (Fig. S5A) and stable total viral RNA levels (Fig. S5B), but markedly skewed positive-sense/negative-sense RNA ratios (Fig. S5C and D). VP1 expression was delayed (Fig. S5E and H), 3C levels were slightly reduced (Fig. S5F), and 3D accumulated to higher levels at late infection stages (Fig. S5G and H). These findings demonstrate that CAE structural integrity is essential for balanced viral gene expression and productive replication.

### CAE structural perturbation reduces virus production through impaired genome replication

**Fig 6:**
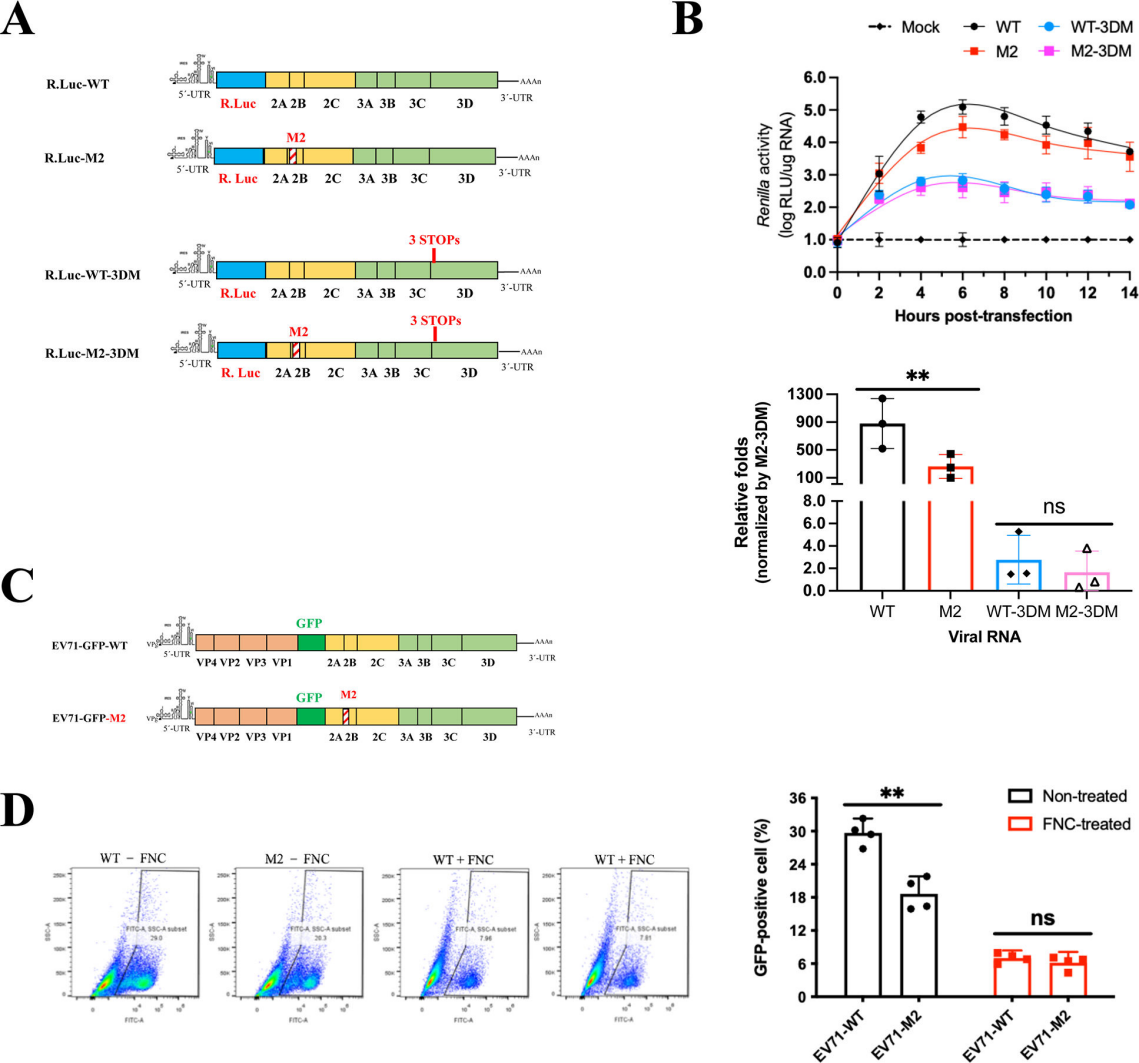
The M2 mutation predominantly impairs viral genome replication. (**A**) Schematic of luciferase reporter replicons derived from EV-A71-WT and EV-A71-M2, in which the P1 capsid-coding region was replaced with *Renilla* luciferase. Corresponding 3D-lethal variants by introducing three in-frame stop codons at the N-terminus. (**B**) Luciferase activity in 293FT cells transfected with RNA species *in vitro-*transcribed from the indicated replicons. The illuminance was detected every 2 h (upper panel). Specifically, the relative levels at 6 h post-transfection were compared with the activity of M2-3DM (lower panel). (**C**) Schematic of GFP reporter viruses generated by inserting *GFP* between *VP1* and *2A*, preserving the C-terminal three amino acids of VP1 and the N-terminal three amino acids of 2A at the junctions to facilitate authentic proteolytic processing. (**D**) Percentage of GFP-positive cells following infection with reporter viruses. RD cells were infected at an MOI of 0.1 for 16 h and then treated with 100 nM azvudine (FNC) or vehicle for 36 h prior to flow cytometric analysis. The left panel shows representative results from two independent experiments performed in duplicate; the right panel shows quantitative data as mean ± SD. **P < 0.01 (Student’s *t*-test).

To determine the stage of the viral life cycle affected by M2 mutations, we first noted that synonymous substitutions do not alter the protein sequence; thus, cell entry and virion release should remain unaffected, leaving genome replication and protein translation as likely targets. We tested this using two complementary systems. First, we generated EV-A71-WT and M2 replicons in which the P1 capsid region was replaced with *Renilla* luciferase and then engineered 3D-lethal variants by introducing three in-frame stop codons at the N-terminus (Fig. 6A; Fig. S8B). Immunostaining confirmed that the 3D-lethal mutation abolished 3D protein expression (Fig. S8C). Following *in vitro* transcription and transfection, luciferase activity was detected every 2 h. The 3D-lethal mutation reduced luciferase activity in both WT and M2 backgrounds, consistent with inhibition of RNA replication (WT vs WT-3DM and M2 vs M2-3DM). Importantly, M2 replicons displayed significantly reduced luciferase activity relative to WT, showing the consistence with that in viral infection (Fig. 5). Notably, the decrease was abolished when viral RNA replication was blocked (WT vs M2 and WT-3DM vs M2-3DM) (Fig. 6B). These results demonstrate that the M2 mutations specifically impair viral RNA replication rather than primary translation of viral RNA.

To confirm this mechanism in the context of infectious virus, we utilized EV-A71 reporter viruses expressing GFP between VP1 and 2A (Fig. 6C). Infected cells were treated with azvudine (FNC; 100 nM), a pyrimidine analog that specifically inhibits the 3D polymerase (29), or left untreated. The M2 mutations significantly reduced the percentage of GFP-positive cells, but this reduction was nearly abolished upon treatment with the 3D inhibitor (Fig. 6D). Collectively, these findings indicate that CAE structural disruption reduces viral yields primarily through defective genome replication.

### CAE structural mutation impairs viral RNA binding to 3D polymerase

**Fig 7:**
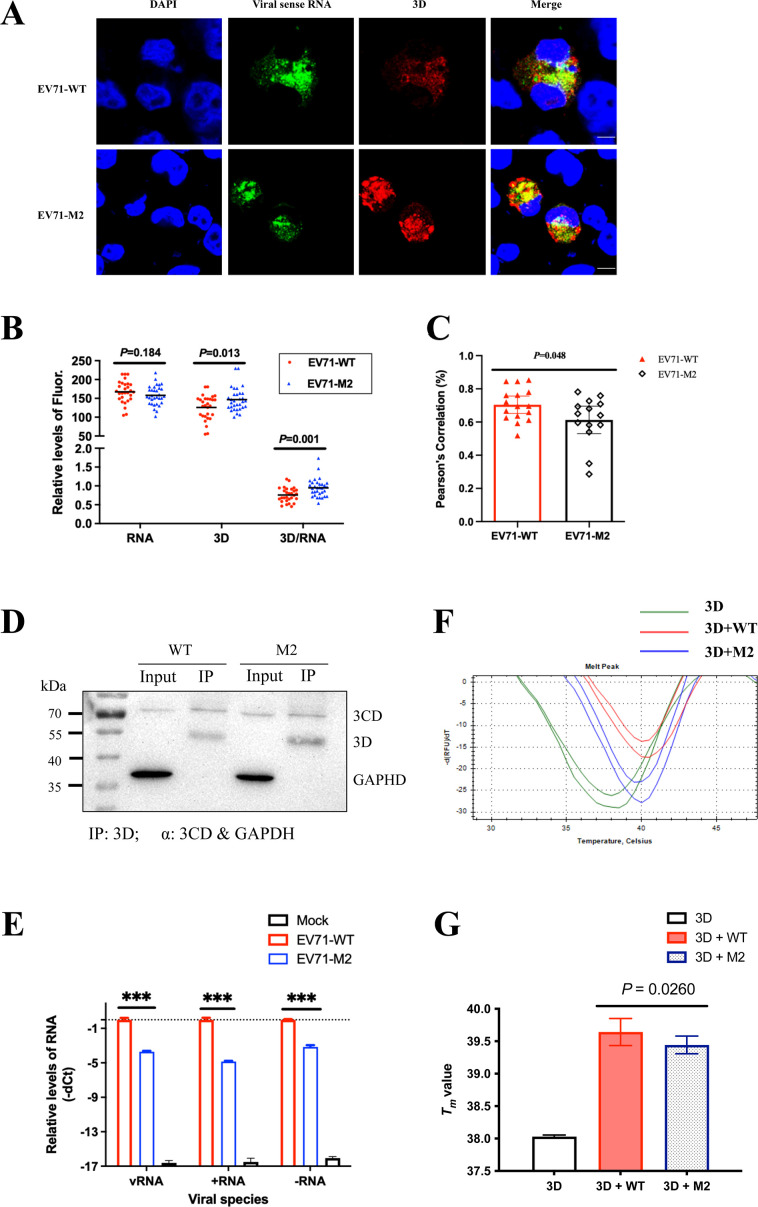
Impact of the M2-Mutation on viral RNA interaction with 3D polymerase. (**A**) Visualization of viral RNA and 3D polymerase by RNA-FISH combined with immunofluorescence. RD cells were infected at an MOI of 10 PFU/cell for 24 h. Viral positive-sense RNA was detected using third-generation hybridization chain reaction (HCR v3.0), followed by staining with anti-3D antibody and Cy3-conjugated secondary antibody. Nuclei were counterstained with DAPI. Confocal images were acquired on a Leica TCS SP5. (**B**) Quantification of 3D and viral RNA fluorescence intensity and the 3D:RNA ratio in individual cells. (**C**) Colocalization analysis of 3D and viral RNA using Fiji (ImageJ v2.5.0). Data represent 16 images (EV-A71-WT) and 14 images (EV-A71-M2) from two independent experiments. Each data point represents a single cell. (**D and E**) CLIP-qPCR analysis of viral RNA binding to 3D. RD cells were infected at an MOI of 1.0 for 16 h, UV-cross-linked (254 nm, 200 mJ/cm²), and lysed. Immunoprecipitation was performed using anti-3D antibody or IgG control. (**D**) Immunoblot for 3D in input and immunoprecipitation (IP) samples. (**E**) qPCR quantification of total, positive-sense, and negative-sense viral RNA in IP samples. Data represent two independent experiments performed in duplicate. (**F and G**) Thermal shift assay (TSA) of 3D–RNA binding. Purified 3D (2 μM) was incubated with Domain II RNA (4 μM; wild-type or M2) and 5× SYPRO Orange. Melting curves (**F**) and *Tm* values (**G**) were determined using a Boltzmann Sigmoid fit in GraphPad Prism. Data represent three independent experiments performed in duplicate. ***P < 0.05 (Student’s *t*-test).

Mutations within the 2A–2B CAE modulate both genome replication kinetics and 3D polymerase accumulation (Fig. 5; Fig. S5). Given that 3D is the viral RNA-dependent RNA polymerase essential for replication, we hypothesized that M2 mutations impair viral RNA–3D interactions. To visualize these complexes, we employed RNA fluorescence *in situ* hybridization (RNA-FISH) using third-generation hybridization chain reaction (HCR v3.0). This method utilizes split-initiator probe pairs to minimize the background and amplify fluorescent signals proportionally to target RNA abundance (30–32). In cells infected with EV-A71-M2, 3D protein levels were elevated compared to wild-type despite comparable viral RNA levels (Fig. 7A and B). However, colocalization between viral RNA and 3D was reduced in EV-A71-M2-infected cells (Fig. 7C), suggesting impaired RNA–protein association. To quantify this interaction, we performed cross-linking immunoprecipitation quantitative PCR (CLIP-qPCR) using UV cross-linking (33, 34). Following the immunoprecipitation of equivalent amounts of 3D polymerase, significantly less viral RNA—particularly positive-sense genomic RNA—was recovered from EV-A71-M2-infected cells compared to wild-type (Fig. 7D and E), confirming reduced RNA-binding affinity.

To characterize this interaction biochemically, we expressed and purified recombinant 3D-GST fusion protein from *E. coli*. Following GST affinity purification and thrombin cleavage, the polymerase was further purified by Benzamidine Sepharose chromatography (to remove thrombin) and HiTrap Q FF anion exchange chromatography, followed by a final GSTrap polishing step (Fig. S6). We assessed the binding between purified 3D and *in vitro-*transcribed Domain II RNA (Fig. 2A) using thermal shift assay (TSA), which detects protein stabilization upon ligand binding as an increase in melting temperature (*Tm*) measured by SYPRO Orange fluorescence (35). Consistent with cellular data, 3D exhibited a greater *Tm* shift upon binding to wild-type RNA compared to M2-mutant RNA (Fig. 7F and G). Collectively, these findings indicate that M2 mutations within the IRES-like CAE reduce the affinity of viral RNA for 3D polymerase.

### Mutations within the IRES-like CAE impair viral fitness

**Fig 8:**
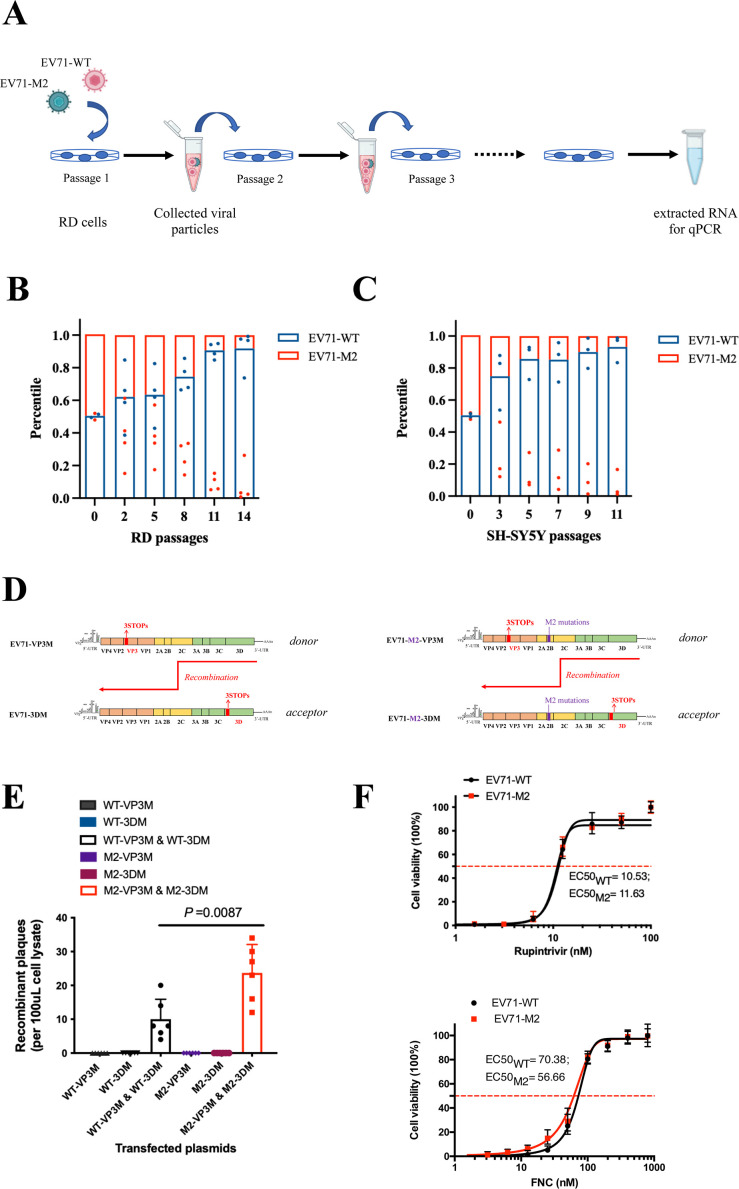
The M2 mutation modulates viral fitness. (**A**) Schematic of the competitive infection assay. (**B and C**) Competitive fitness in RD (**B**) and SH-SY5Y (**C**) cells. Cells were co-infected with EV-A71-WT and EV-A71-M2; viral RNA was quantified at each passage by allele-specific qPCR. Data represent the percentage of WT- or M2-genome copies relative to total viral genomes. (**D**) Schematic of the recombination assay using lethal mutant clones. EV-A71-VP3M (lethal in VP3) and EV-A71-3DM (lethal in 3D) were co-transfected; viable recombinants were rescued by template-switching. (**E**) Recombination frequency determined by plaque assay. Complementary lethal clones (2 μg each) were co-transfected into T7 polymerase–expressing cells. At 48 hpt, cells were subjected to three freeze-thaw cycles and progeny titers determined by plaque assay. Data represent three independent experiments performed in duplicate. (**F**) Drug susceptibility assays. RD cells were infected with EV-A71-WT or EV-A71-M2 at an MOI of 0.1 and treated with serial dilutions of rupintrivir (3C protease inhibitor) or azvudine (FNC; 3D polymerase inhibitor). Cell viability was measured at 72 hpi using the CCK-8 assay. EC₅₀ values were calculated by nonlinear regression. Data represent three independent experiments performed in quintuplicate. *P* < 0.05 (Mann-Whitney U test).

To assess whether CAE structural perturbation affects viral fitness, we performed competition assays between EV-A71-WT and EV-A71-M2. RD cells were co-infected with both viruses, and progeny were serially passaged for 15 generations. Viral RNA was quantified at each passage using virus-specific qPCR (Fig. 8A; Fig. S7A through C). EV-A71-M2 exhibited a progressive fitness cost, with its proportion declining continuously; by passage 11, the mutant was virtually outcompeted by wild-type (Fig. 8B). A similar trend was observed in SH-SY5Y cells, where the M2 mutant dropped below 10% of the population by passage 9, indicating a more severe fitness defect in neuronal cells (Fig. 8C).

Viral recombination via RdRp-mediated template switching can restore infectivity following lethal mutations and serve as a fitness indicator. To determine whether CAE structure modulates the recombination frequency, we employed a classic marker rescue assay using two lethally mutated clones: EV-A71-VP3M (containing three stop codons in VP3) and EV-A71-3DM (containing three stop codons at the 3D N terminus) (Fig. 8D; Fig. S8A). Protein synthesis was abolished at the respective mutation sites, confirming lethality (Fig. S8C). Co-transfection of these clones yielded infectious recombinant progeny (detected by VP1 immunostaining of infected RD cells) (Fig. S8D). Importantly, when the M2 mutation was introduced into both lethal clones (EV-A71-M2-VP3M and EV-A71-M2-3DM), the recombination frequency increased significantly compared to that of wild-type controls, as quantified by plaque assay (Fig. 8E). It suggested that CAE structural disruption enhances recombination efficiency.

High drug resistance can enhance viral replication in a detrimental environment and is considered another indicator of viral fitness. To evaluate the impact of CAE structural perturbation on antiviral susceptibility, we determined EC₅₀ values for rupintrivir (AG7088; 3C protease inhibitor) (36) and azvudine (FNC; 3D polymerase inhibitor) (29). While rupintrivir susceptibility was comparable between EV-A71-M2 (11.63 nM) and wild-type (10.53 nM), the M2 mutant was significantly more susceptible to FNC (EC₅₀: 56.66 nM versus 70.38 nM for wild-type) (Fig. 8F). These data indicate that CAE structural integrity specifically contributes to resistance against 3D-targeting antivirals.

## DISCUSSION

Cis-acting elements (CAEs) are essential RNA structures that regulate nearly every stage of the viral life cycle. In positive-sense RNA viruses, these elements are critical for viral gene expression, such as IRESes and 3′-CITEs (1). Some CAEs also orchestrate the switch from translation to replication and guide the synthesis of genomic RNAs. In picornaviruses, well-characterized CAEs include the 5′-cloverleaf (OriL), the cis-acting replication element (CRE/OriI) within the 3C coding region, and OriR within the 3′ UTR. However, few CAEs have been identified within the coding regions of picornaviruses beyond OriI (37). In this study, using a bicistronic reporter system for genome-wide screening (Fig. 1), we identified several IRES-like CAEs within the EV-A71 coding region, consistent with recent predictions (10). These elements exhibited weak IRES activity and were distributed non-uniformly, clustering within the VP3–VP1 and 2A–2B regions (Fig. 1; Fig. S2). Notably, this distribution is consistent with ribosome profiling data demonstrating ribosome accumulation within these regions (38), suggesting that these CAEs may recruit or stall ribosomes to facilitate translational re-initiation or coordinate the transition from translation to RNA synthesis (39, 40).

We characterized the CAE within the 2A–2B region, demonstrating that it can drive downstream reporter expression in transfected cells and in rabbit reticulocyte lysates (Fig. 1E, 2B and 4C). Domain mapping identified nt 3,730–3,986 as the primary regulatory region (Fig. 2B). The functional importance of RNA structure was confirmed using synonymous mutations (M2 type), which significantly altered secondary structure and abrogated regulatory activity (Fig. 2C). Using a high-efficiency reverse genetics system coupled with fluorescent reporters, we observed GFP expression from the 3D locus even when 5′-IRES-mediated translation was blocked by lethal VP3 mutations or IRES deletion (Fig. 3 and 4). According to our knowledge, it was the first time to demonstrate that the EV-A71 genome can be translated via a 5′-IRES-independent mechanism. While Kim et al. (26) previously proposed IRES-independent translation based on indirect evidence linking recombination to translation, our study directly demonstrates this phenomenon. IRES-independent clones bearing an RFP substitution of the VP3–VP1 element lacked detectable RFP expression from the VP1 locus but retained GFP expression from the 3D locus (Fig. 3C and 4), indicating that residual 3D expression specifically depends on the 2A–2B CAE rather than the VP3–VP1 element. This conclusion was validated by the M2 mutations, which significantly impaired 5′-IRES-independent translation (Fig. 4D).

Furthermore, M2 synonymous mutations in the rescued virus reduced viral yields and positive-sense RNA synthesis while unexpectedly increasing negative-sense RNA and 3D polymerase accumulation at later time points (Fig. 5; Fig. S5). The disproportionate decrease in positive-sense RNA relative to negative-sense RNA suggests specific impairment of positive-strand RNA synthesis initiation from the negative-sense template—an unexpected function for an element related to IRES activity. This may reflect M2 mutation-induced RNA structural remodeling, potentially generating stable secondary structures in the negative-sense strand that cause premature termination during negative-strand synthesis. We also noted that VP1 and 3D levels in M2 variant-infected cells showed significant alterations at different viral stages but did not follow the same trends. The mechanism underlying altered relative proportions of VP1 and 3Dpol produced from the same polyprotein remains incompletely understood. Multiple mechanisms may contribute, including host factor-mediated differential degradation and variant-specific translational initiation independent of the canonical 5′-IRES, such as the IRES-like element-driven 3D translation we observed. We hypothesize that elevated 3D expression might partially compensate for reduced genomic RNA synthesis. Using luciferase reporter replicons and GFP reporter viruses, we confirmed that M2 mutations impair viral genome replication (Fig. 6). Though the 2A–2B CAE exhibits only ~20% of the activity of 5′-IRES, our findings reveal a distinct role in modulating replication complex function.

To investigate the mechanistic basis, we examined viral RNA–3D interactions using RNA-FISH combined with immunofluorescence. While 3D protein levels were higher in M2-infected cells during mid-to-late infection (consistent with immunoblotting; Fig. 5G; Fig. S5G), colocalization with viral RNA was reduced compared to that of the wild-type (Fig. 7C). CLIP-qPCR confirmed that 3D bound fewer copies of viral RNA—particularly positive-sense RNA—in M2-infected cells (Fig. 7E). Thermal shift assays demonstrated reduced binding affinity between purified 3D and M2-mutant RNA relative to the wild-type (Fig. 7G). Collectively, these data indicate that CAE structural perturbation impairs viral RNA–polymerase interactions, reducing replication efficiency despite compensatory 3D upregulation. The M2 mutations likely alter higher-order RNA genome structure, as detected by SHAPE, thereby reducing RNA remodeling efficiency and binding affinity to 3D polymerase rather than directly interacting with the polymerase.

Synonymous mutations can modulate virulence, drug resistance, and host interactions, serving as drivers of viral evolution (41–45). To determine fitness consequences, we performed competition assays. M2 virus was rapidly outcompeted by the wild-type in both RD and SH-SY5Y cells, with more rapid elimination in neuronal cells (Fig. 8B and C). This disparity likely reflects cell-type-specific differences in host factor availability or viral kinetics that exacerbate the replication defect. RNA recombination, mediated by RdRp-dependent template switching, enhances viral adaptability (46, 47). Unexpectedly, M2 mutations significantly increased recombination frequency (Fig. 8E), potentially by reducing RNA–polymerase affinity and facilitating premature template release and re-association. However, this advantage did not compensate for the fitness cost as M2 virus remained at a competitive disadvantage. Drug susceptibility assays revealed equivalent sensitivity to the 3C inhibitor rupintrivir but significantly increased susceptibility to the 3D inhibitor FNC in M2 mutants (Fig. 8F), consistent with impaired polymerase function.

Here, we demonstrate that EV-A71 translation occurs independently of the 5′-IRES. Genome-wide screening identified IRES-like CAEs within the coding region; the 2A–2B element specifically mediates 5′-IRES-independent translation of the 3D locus. Our findings establish that CAE RNA structure is a critical determinant of viral fitness, modulating polymerase binding, replication efficiency, and evolutionary dynamics. While the effects of these synonymous mutations in animal models remain to be determined and the conservation of such elements in related enteroviruses (e.g., coxsackieviruses A6, A10, and A16) requires investigation, our study illuminates a previously unappreciated layer of regulatory complexity in picornavirus genome function.

## MATERIALS AND METHODS

### Virus strain, cell lines, and reagents

Enterovirus A71 (EV-A71) strain 064-Shanghai (GenBank accession number HQ891927) was isolated from a patient with hand, foot, and mouth disease, as described previously (48). Rhabdomyosarcoma (RD), HEK293FT and SH-SY5Y (human neuroblastoma), and COS-7 cells were obtained from the American Type Culture Collection (ATCC) and maintained in Dulbecco’s modified Eagle medium (DMEM; Gibco) supplemented with 10% fetal bovine serum (FBS). Cell lines stably expressing T7 polymerase, including 293FT/T7pol and COS-7/T7pol, were generated as previously described (27). Mouse monoclonal antibodies specific for β-actin and GAPDH were purchased from Cell Signaling Technology; anti-EV-A71 VP1 (MAB1225-M05) from Abnova; and anti-3C, anti-3D, and anti-3CD antibodies from GeneTex. Oligonucleotides for split-initiator probes and Alexa Fluor 488–labeled hairpin amplifiers for RNA-FISH were synthesized by Thermo Fisher Scientific (Shanghai).

### Plasmid construction and screening for cis-acting elements

To generate the bicistronic reporter plasmid pMSCV-*GFP*-Insert-*RFP*, we modified pMSCV-IRES-mCherry FP (Addgene #52114, a gift from Dario Vignali). The GFP coding sequence, followed by two stop codons, was inserted between the murine stem cell virus (MSCV) promoter and the encephalomyocarditis virus (EMCV) IRES using Gibson assembly with *Eco*RI and *Bam*HI. The EMCV IRES was subsequently replaced with test sequences, including the cytomegalovirus (CMV) promoter, the EV-A71 5′ UTR (nt 1–743), or EV-A71 IRES deletion mutants (ΔSLII: Δnt 129–167; ΔSLIV: Δnt 445–561). To screen for CAEs within the viral genome, the EV-A71 coding region was tiled with 21 overlapping fragments (~600–700 nt each). Plasmids (3 μg per well) were transfected into HEK293FT cells in 6-well plates. GFP and RFP expression was quantified by flow cytometry or microscopic enumeration at specified time points.

### Luciferase reporter assays

To generate bicistronic luciferase reporters, GFP and RFP in pMSCV-*GFP*-Insert-*RFP* were replaced with *Renilla* luciferase (*Rluc*) and firefly luciferase (*Fluc*), respectively, creating pMSCV-*Rluc*-Insert-*Fluc*. The EMCV IRES was replaced with the EV-A71 5′ UTR (nt 1–743) or candidate CAE sequences. HEK293FT cells in 24-well plates were transfected with 800 ng reporter plasmid per well using Lipofectamine 2000 (Invitrogen) according to the manufacturer’s protocol. Each condition was performed in triplicate and repeated in at least three independent experiments. Dual-luciferase activities were measured 24–48 h post-transfection using the Dual-Luciferase Reporter Assay System (Promega). For RNA transfection assay, *Renilla* luciferase activity was monitored every 2 h post-transfection using EnduRen Live Cell Substrate (Promega) as per the manufacturer’s protocol. Luminescence was measured on a Tecan Infinite M1000 with 1,000 ms integration.

### SHAPE assay and RNA structure analysis

Selective 2′-hydroxyl acylation analyzed by primer extension and mutational profiling (SHAPE-MaP) was performed as described (23, 49). Briefly, viral RNA was gently extracted from EV-A71-WT- or M2-infected cells and allowed to refold *in vitro*. RNA was then treated with N-methylisatoic anhydride (NMIA) or DMSO (control) and reverse-transcribed using gene-specific primers (5′-TGAGAGCTTCAACCTCCCTTG-3′). cDNA was used for PCR amplicon library preparation and high-throughput sequencing. SHAPE reactivity profiles were generated using ShapeMapper 2, and secondary structure modeling was performed with RNAfold (v2.4.17).

### Plaque assay

The plaque assay was conducted according to our previously described methodology (48). RD cells were seeded in 6-well plates at 5 × 10⁵ cells/well and incubated for 16 h. Monolayers were infected with 0.5 mL of serially diluted virus for 1 h, washed with PBS, and overlaid with 2 mL DMEM containing 2% FBS and 0.25% low-melting-point agarose. After 72 h, plaques were stained with 0.1% crystal violet in 4% formaldehyde for 2–4 h and counted. Titers were calculated as the geometric mean ± standard deviation from at least three independent assays.

### Quantitative RT-PCR

Total RNA (1 μg) was reverse-transcribed using random primers and Moloney murine leukemia virus (M-MLV) reverse transcriptase (42°C, 45 min; heat-inactivated). EV-A71 cDNA was amplified using gene-specific primers (95°C, 4 min; 40 cycles of 95°C, 5 s; 60°C, 10 s) on an Applied Biosystems 7500 Fast Real-Time PCR System. *β-actin* served as the endogenous control. Relative quantification used the ΔΔCt method. For strand-specific quantification, reverse transcription was primed with T7- or SP6-tagged primers (50), followed by standard qPCR (Table S1). For competition assays, allele-specific primers targeting the M2 mutation site were employed (Fig. S7A).

### Immunoblotting

Cells were lysed on ice in RIPA buffer supplemented with protease and phosphatase inhibitors (PMSF, leupeptin, and aprotinin). Lysates were resolved by SDS-PAGE and transferred to PVDF membranes. Membranes were incubated with primary antibodies against target proteins and β-actin or GAPDH (loading controls), followed by horseradish peroxidase–conjugated anti-mouse secondary antibody (1:5,000). Signals were detected using enhanced chemiluminescence (ECL; Amersham Biosciences). To ensure accurate loading and internal control assessment, antibodies against β-actin or GAPDH were co-incubated with the primary antibodies targeting proteins of interest, thereby minimizing variations due to experimental manipulation. This protocol was consistently followed for the detection of all corresponding target proteins.

### RNA-FISH combined immunofluorescence (IMF) staining for microscopy

RD cells were seeded in 35-mm glass-bottom dishes (2 × 10⁵ cells/dish) and infected with EV-A71-WT or EV-A71-M2 at an MOI of 1 PFU/cell for 24 h. RNA-FISH was performed using third-generation hybridization chain reaction (HCR v3.0) as described (30), with modifications. Cells were fixed in 4% paraformaldehyde in PBS, permeabilized with 0.1% Tween 20 in PBS (PBST) for 10 min at room temperature, and prehybridized in 30% probe hybridization buffer (300 μL/well) for 30 min at 37°C. Following washes in 5× SSCT, cells were hybridized overnight at 37°C with 15 pairs of split-initiator probes specific for EV-A71 (1.67 pmol/probe; Table S2). Fluorescent signals were amplified using Alexa Fluor 488–labeled hairpin amplifiers (20 pmol; Table S2). Cells were then stained with anti-3D mouse monoclonal antibody, followed by Cy3-conjugated secondary antibody and counterstained with DAPI. Images were acquired on a Leica TCS SP5 confocal microscope. Fluorescence intensity ratios and colocalization analysis were performed using Fiji (ImageJ v2.5.0).

### CLIP-qPCR

RD cells (3 × 10⁶ cells/10-cm dish) were infected with EV-A71-WT or EV-A71-M2 at an MOI of 1 for 16 h. Following washing with cold PBS, cells were UV-irradiated (254 nm, 200 mJ/cm²) to cross-link RNA–protein complexes. Cells were lysed in RIPA buffer containing protease inhibitor cocktail, PMSF (5 mM), DTT (1.5 mM), and RNase inhibitor (25 U/μL; Takara). Lysates were sonicated (5 s on/5 s off, 40% amplitude, 2 min total, 4°C) and cleared by centrifugation. Supernatants (1 mL) were incubated with polyclonal anti-3D antibody (20 μL) overnight at 4°C, followed by addition of 50 μL Protein A/G magnetic beads (Pierce) for 2 h. Beads were washed and divided equally: one aliquot was analyzed by immunoblotting for 3D immunoprecipitation efficiency; the other was processed for RNA extraction using TRIzol, followed by qPCR to quantify total, positive-sense, and negative-sense viral RNA.

### Expression, purification of 3D polymerase, and thermal shift assay

The EV-A71 3D coding sequence was subcloned into pGEX-4T. *E. coli* BL21(DE3) cells were transformed and protein expression induced with 0.2 mM IPTG at 30°C for 10 h. GST-3D fusion protein was purified on a GSTrap 4B column (Cytiva) and eluted in 50 mM Tris-HCl (pH 8.0), 10 mM reduced glutathione, and 1 mM DTT. The GST tag was removed by thrombin cleavage (Beyotime), and 3D protein was further purified by benzamidine-Sepharose (to remove thrombin) followed by anion exchange chromatography (HiTrap Q FF, Cytiva). Purity (>99%) was confirmed by SDS-PAGE with Coomassie blue staining. For thermal shift assays (TSA), *in vitro*–transcribed CAE Domain II RNA (nt 3730–3986; T7 promoter–driven) was generated using the RiboMAX Large-Scale RNA Production System (Promega). Purified 3D (2 μM) was incubated with RNA (4 μM) in 50 mM Tris-HCl containing 5× SYPRO Orange for 30 min at 4°C. Melting curves were generated by heating samples from 4°C to 95°C (0.5°C/min ramp) in a Bio-Rad CFX96 Touch Real-Time PCR Detection System. *Tm* values were determined by fitting data to a Boltzmann Sigmoid curve using GraphPad Prism.

### Viral recombination assay

Two pairs of lethally mutated infectious clones were generated based on the EV-A71-WT cDNA clone (27): EV-A71-VP3M contained three premature stop codons in VP3 (nt 1726, 1731, and 1753), while EV-A71-3DM contained three stop codons in 3D (nt 5950, 5964, and 5967) (Fig. S8A). Corresponding M2-mutant lethal clones (EV-A71-M2-VP3M and EV-A71-M2-3DM) were similarly constructed. Complementary lethal clones were co-transfected (2 μg each) into T7 polymerase–expressing cells in 6-well plates. At 48 h post-transfection, cells were subjected to three freeze-thaw cycles, and released virus titers were determined by plaque assay.

### Virus drug resistance assay

RD cells (1 × 10⁴ cells/well in 96-well plates) were infected with EV-A71-WT or EV-A71-M2 at an MOI of 0.1 for 1 h and then treated with serial dilutions of rupintrivir (AG7088) or azvudine (FNC). At 72 hpi, the cell viability was measured using the CCK-8 kit according to the manufacturer’s protocol. Drug concentrations reducing cell viability by 50% (EC₅₀) were calculated using nonlinear regression (log[inhibitor] vs normalized response) in GraphPad Prism.

### Statistical analysis

Data were analyzed using GraphPad Prism 10.0. Comparisons between two groups were performed using Student’s *t*-test (two-tailed, unpaired unless otherwise stated). Statistical significance was defined as *P* < 0.05.

## Data Availability

The data that support the findings of this study are presented in the article and its supplemental material. The data are available from the corresponding author upon reasonable request.

## SUPPLEMENTAL MATERIAL

Supplemental figures jvi.00355-26-s0001.pdf Fig. S1 to S8.

Table S1 jvi.00355-26-s0002.docx Primers for EV-A71 qPCR.

Table S2 jvi.00355-26-s0003.xlsx EV-A71 RNA-FISH probe sets.

ASM does not own the copyrights to Supplemental Material that may be linked to, or accessed through, an article. The authors have granted ASM a non-exclusive, world-wide license to publish the Supplemental Material files. Please contact the corresponding author directly for reuse.
