# Supplementary material for: An IRES-like cis-acting element located within the EV-A71 coding region drives translation independent of the 5′-IRES and modulates viral fitness through regulated binding of viral RNA to 3D polymerase
Source: J Virol. 2026 May 27;100(6):e00355-26. doi: 10.1128/jvi.00355-26 (PMC13288635; doi:10.1128/jvi.00355-26)
Supplement: Supplemental figures — Fig. S1 to S8. [file jvi.00355-26-s0001.pdf]

Supporting information

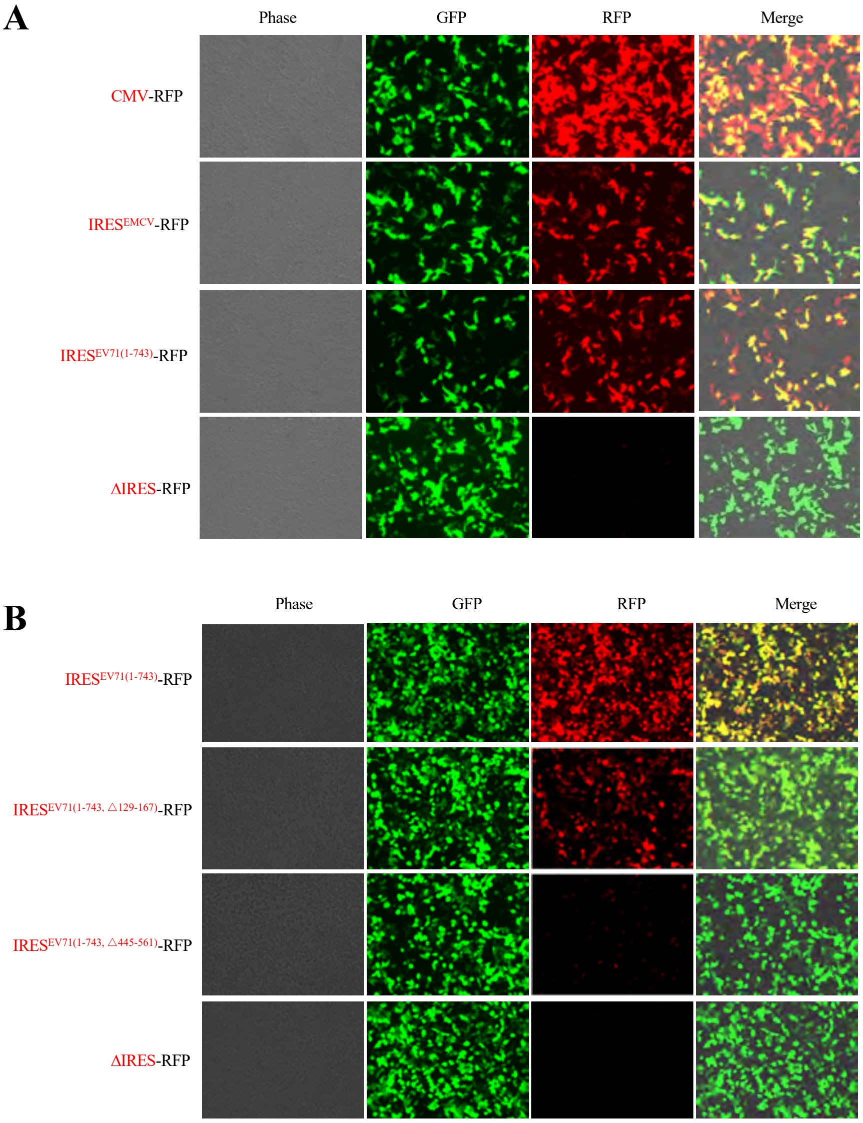

Fig. S1 Preliminary assessment of the bicistronic reporter plasmid pMSCV-GFP-Insert-RFP for screening cis-acting translational elements, related to Fig. 1.

(A) Testing promoter and IRES function. The CMV promoter or viral IRES elements were inserted into the dual-reporter plasmid. Plasmids (0.5 µg/well, 6-well plate) were transfected into 293FT cells for 48 hours and imaged at 100× magnification. Quantified data are shown in Fig. 1B.

(B) Assessment of EV-A71 IRES deletion mutants. The wild-type 5′-UTR or deletion mutants were cloned into the reporter as in (A). Quantified data are shown in Fig. 1C.

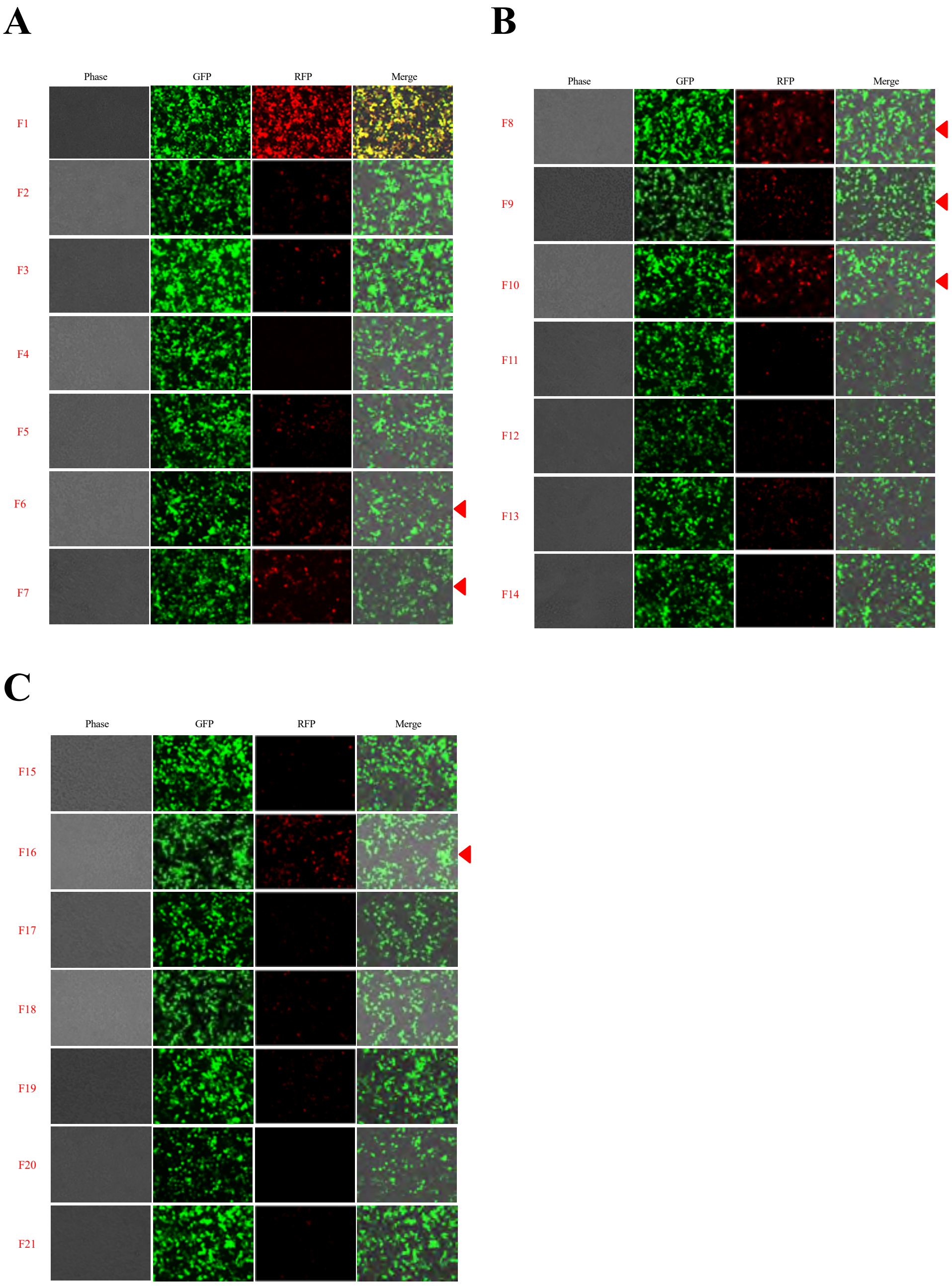

Fig. S2 Comprehensive screening of the EV-A71 genome to identify IRES-like elements, related to Fig. 1. **(A-C)** RFP expression following insertion of EV-A71 fragments F1–F7 (A), F8–F14 (B), and F15–F21 (C). Overlapping ~700 bp cDNA fragments were cloned into pMSCV-GFP-Insert-RFP and transfected into 293FT cells (2 μg/well, 6-well plate) for 72 hours. Representative images from three independent experiments are shown.

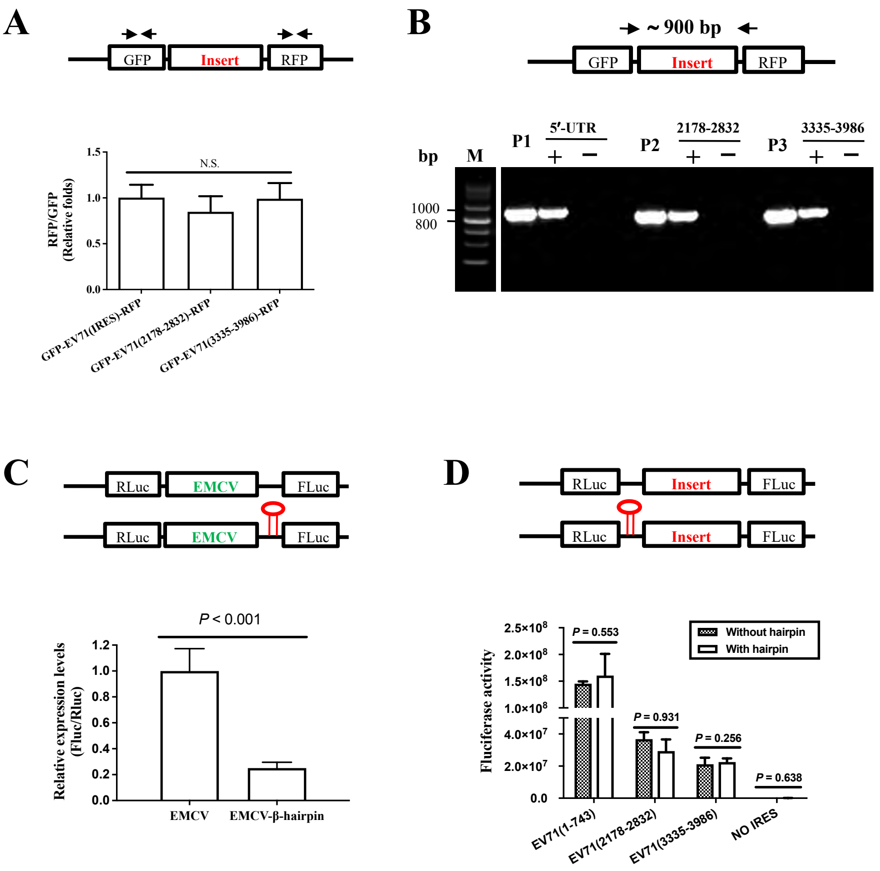

Fig. S3 Validation of IRES-like activity of EV-A71 cDNA fragments in the bicistronic reporter plasmid, related to Fig. 1.

(A) qPCR analysis of GFP and RFP RNA levels. RNA was extracted from cells transfected with reporter plasmids containing the indicated inserts. Values were normalized to the EV-A71 5′ UTR control (set to 1.0). Three independent experiments were performed in duplicate. Arrows indicate primer locations.

(B) RT-PCR detection of transcript integrity to confirm the absence of cryptic splice sites. Primers within GFP and RFP sequences were used to amplify cDNA from the transfected plasmids containing the 5′-UTR, F7 (nt 2178–2832), or F10 (nt 3335–3986). Reactions were performed with (+) or without (−) reverse transcriptase (RT). P1-P3 represent reporter plasmids with 5'-UTR, F7, and F10 insertions, respectively. The presence or absence of reverse transcriptase during RT-PCR is denoted by "+" and "–".

(C) Hairpin control to block ribosomal read-through. A stable stem–loop structure (ΔG = −267 kcal/mol) was inserted upstream of the EMCV IRES in a *Renilla*-firefly luciferase reporter.

(D) Insertion of the hairpin between *Renilla* and firefly luciferase does not inhibit firefly expression. It confirmed that luciferase activity results from internal initiation at the F7 (nt 2178–2832) or F10 (nt 3335–3986) inserts rather than ribosomal read-through. ΔIRES indicates the empty vector control.

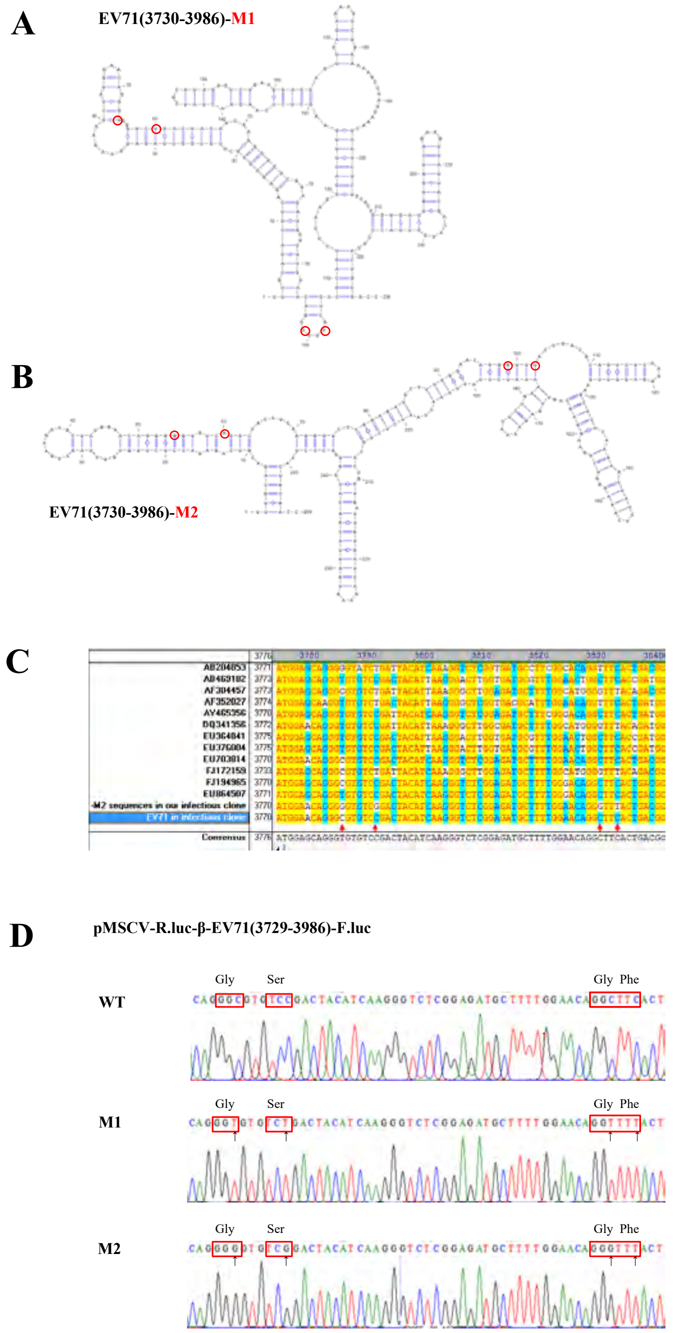

Fig. S4 RNA structure modeling of the IRES-like CAE and sequence validation, related to Fig. 2.
(A and B) Predicted secondary structures of Domain II containing M1 (A) or M2 (B) synonymous mutations. In M1, cytosines at positions 54, 60, 99, and 102 of the CAE (corresponding to genome positions 3782, 3788, 3827, and 3830) were mutated to thymine. In M2, positions 54, 60, and 99 were mutated to guanine, and position 102 to thymine. Mutated nucleotides are highlighted in red.
(C) Prevalence of M2-type synonymous mutations in clinical isolates. The wild-type is highlighted at left; arrows indicate mutated positions.
(D) Sequencing validation of M1 and M2 mutations in luciferase reporter plasmids. Red boxes indicate codons containing synonymous mutations; arrows mark mutation sites.

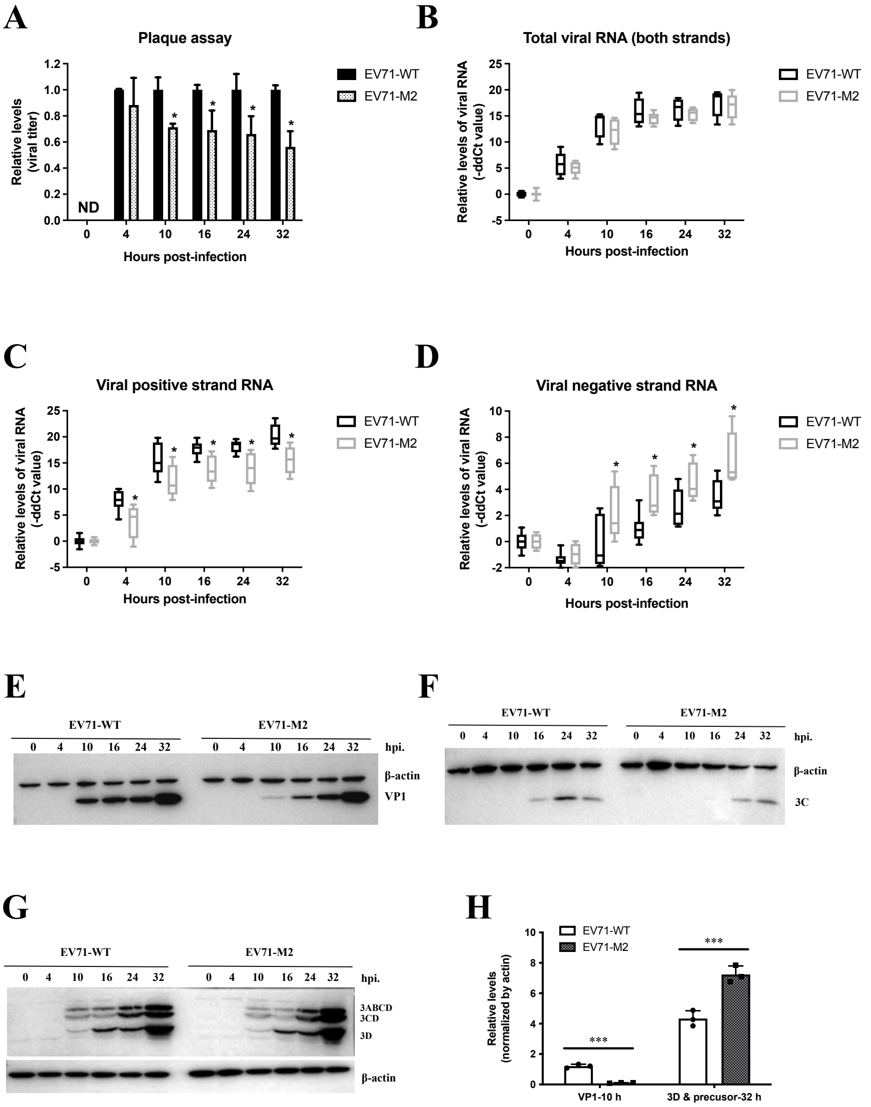

Fig. S5 Comparative replication kinetics of EV-A71-M2 and EV-A71-WT in SH-SY5Y cells, related to Fig. 5.

(A) Viral titers in culture supernatants determined by plaque assay. SH-SY5Y cells were infected at an MOI of 20 PFU/cell, and supernatants were harvested at the indicated times.

(B) qPCR to quantify total viral genome copy numbers.

(C and D) Positive-sense (C) and negative-sense (D) viral RNA quantified by strand-specific qPCR.

(E–G) Immunoblot detection of VP1 (E), 3C (F), and 3D (G). β-actin or GAPDH served as loading controls.

(H) Quantification of VP1 and 3D protein levels from panels (E) and (G). Data represent mean ± standard deviation from three independent experiments. *$P < 0.05$, ***$P < 0.001$ (Student's *t*-test).

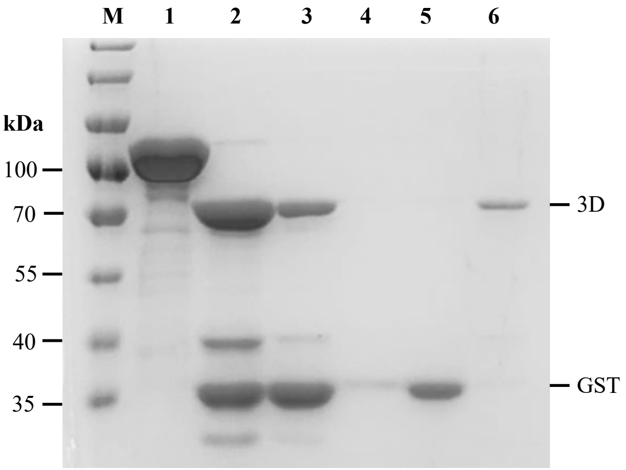

M: Protein marker;

1. Primarily purified GST-3D;

2. GST-3D cleaved by thrombin;

3. Washed thrombin;

4. Low salt buffer washed GST;

5. High salt buffer washed GST;

6. Purified 3D elution.

Fig. S6 Purification of recombinant 3D polymerase, related to Fig. 7.

Coomassie brilliant blue staining of purified 3D protein fractions. GST-3D fusion protein was expressed in *E. coli*, purified on GSTrap 4B (lane 1), cleaved with thrombin (lane 2), and subjected to benzamidine-Sepharose (lane 3) and HiTrap Q FF anion exchange chromatography washed by low salt buffer (lane 4) and high salt buffer (lane 5). Lane 6 shows the final purified 3D after GSTrap polishing; M shows molecular weight markers.

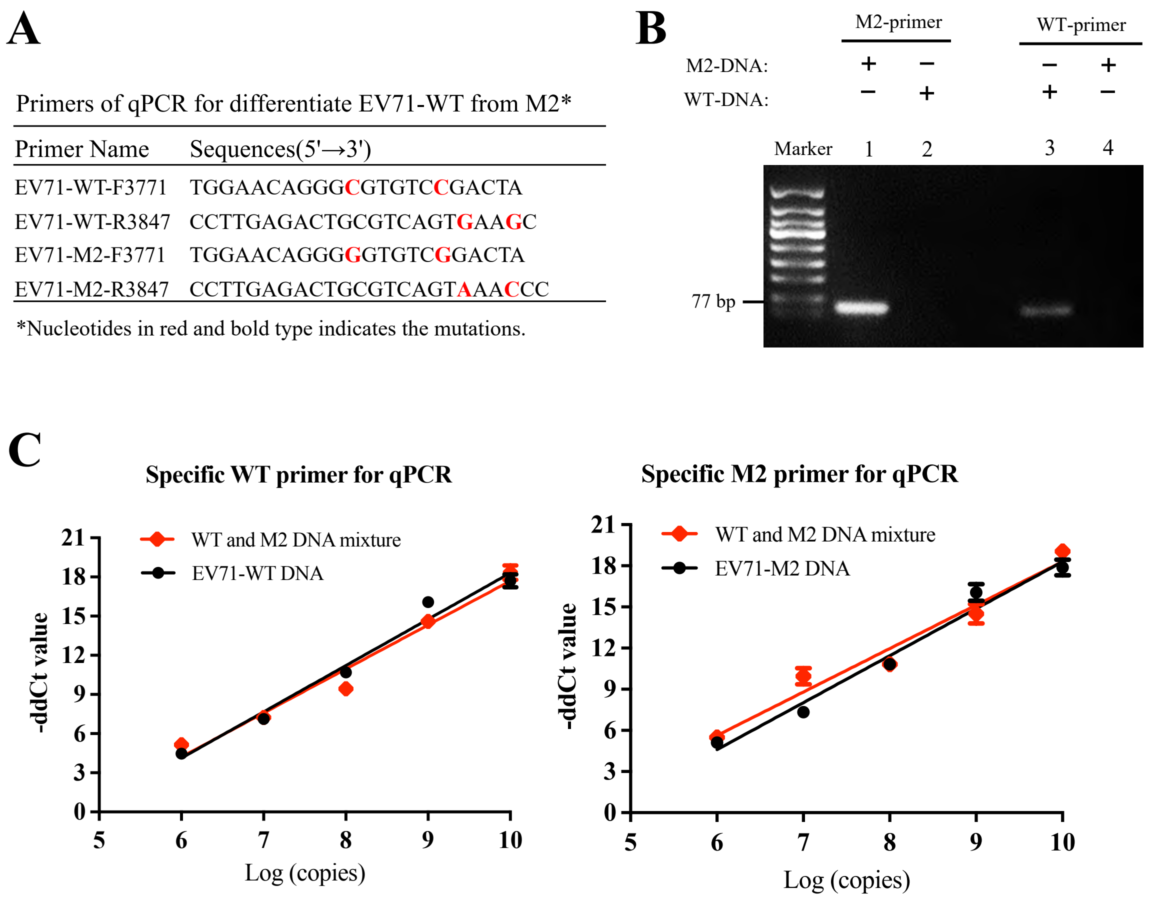

Fig. S7 Optimization of allele-specific qPCR for competitive fitness assays, related to Fig. 8.

(A) Primer design for discrimination of EV-A71-WT and EV-A71-M2 genomes. M2-specific primers contain two nucleotide mismatches (highlighted in red) to ensure specific amplification.

(B) Specificity validation by agarose gel electrophoresis. DNA from EV-A71-WT or EV-A71-M2 served as template for qPCR with the indicated allele-specific primers.

(C) Standard curves for EV-A71-WT (left) and EV-A71-M2 (right) qPCR demonstrating specificity in mixed template reactions. Standard curves were generated independently for each experiment; a representative example is shown.

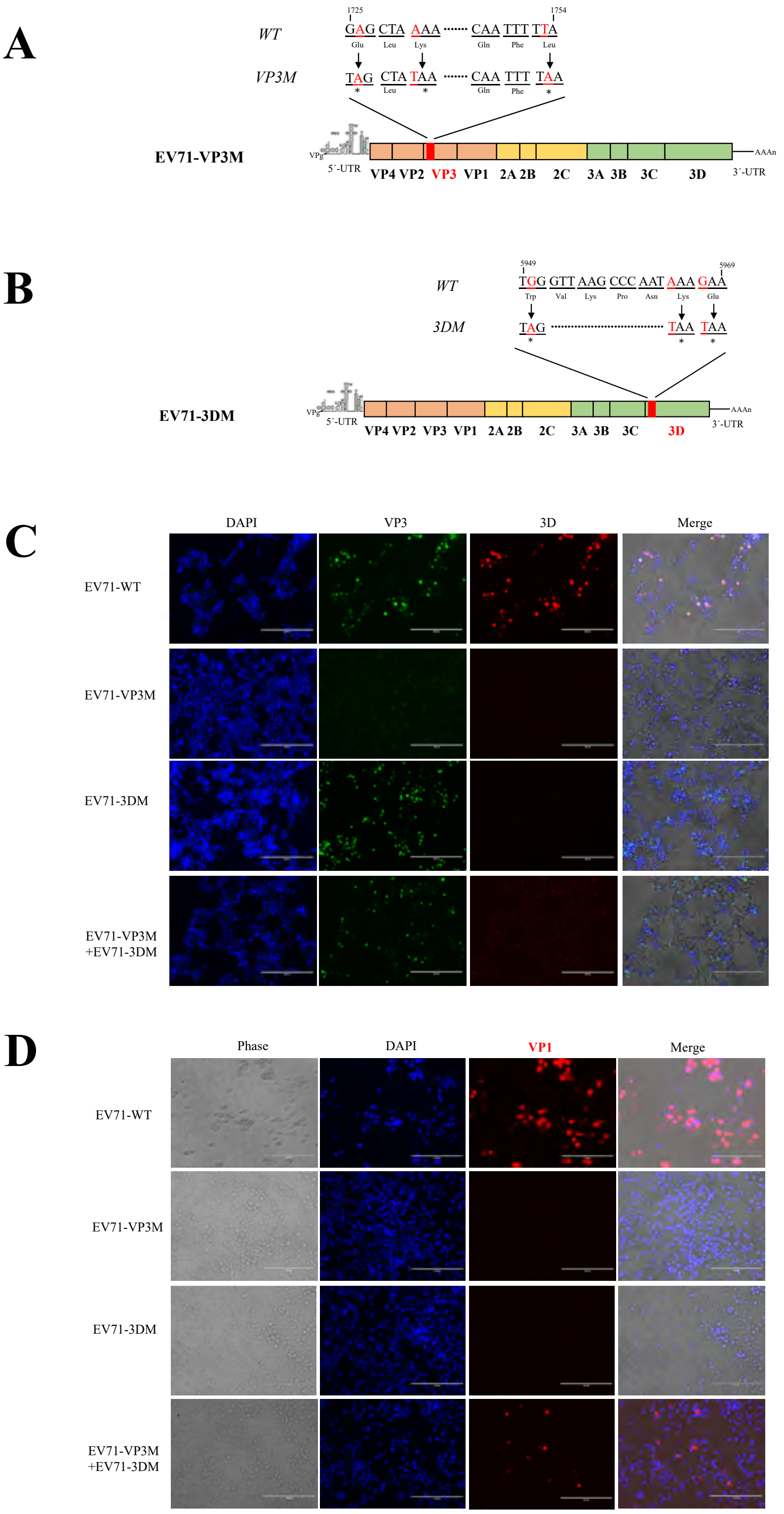

Fig. S8 Lethal mutant clones for recombination assays, related to Fig. 8.

(A and B) Schematics of lethal mutants. (A) EV-A71-VP3M contains three premature stop codons in the 5′ region of VP3 (nt 1726, 1731, and 1753). (B) EV-A71-3DM contains three premature stop codons in the 5′-region of 3D (nt 5950, 5964, and 5967).

(C) Immunofluorescence of 293FT/T7pol cells transfected with the indicated lethal clones (1 µg/well, 24-well

plate) and fixed at 24 hpt. VP3 (green) and 3D (red) were detected using monoclonal antibodies; nuclei were counterstained with DAPI.

(D) Rescue of infectious virus by recombination. Culture supernatants from the transfections in (C) were used to infect RD cells; cells were fixed at 24 hpi and stained for VP1 (green) and nuclei (DAPI).
