# Supplementary material for: An IRES-like cis-acting element located within the EV-A71 coding region drives translation independent of the 5′-IRES and modulates viral fitness through regulated binding of viral RNA to 3D polymerase
Source: J Virol. 2026 May 27;100(6):e00355-26. doi: 10.1128/jvi.00355-26 (PMC13288635; doi:10.1128/jvi.00355-26)
Supplement: Table S1 — Primers for EV-A71 qPCR. [file jvi.00355-26-s0002.docx]

**Table S1 Primers for EV-A71 quantitative RT-PCR**

| **Objective** | **Primer name** | **Function** | **Sequence (5' to 3')** |
| --- | --- | --- | --- |
| For standard qPCR | 3A-5192F | Forward | tatccctgaaactcccacca |
|  | 3A-5284R | Reverse | aacgagacaaccgccactac |
|  | β-actin-F | Forward | gaagtaccccatcgagcacg |
|  | β-actin-R | Reverse | ggatagcacagcctggatagca |
| For positive-stranded RNA qPCR | SP6-R3893 | Reverse transcription | acgcgtatttaggtgacactatagtgctccttcagaccctatga |
|  | SP6-R | Forward | acgcgtatttaggtgacactatag |
|  | WT-F3774 | Reverse for WT | gaacagggcgtgtccgacta |
|  | M2 F3774 | Reverse for M2 | gaacagggggtgtcggacta |
| For negative-stranded RNA qPCR | T7-F3644 | Reverse transcription | aggtaatacgactcactatagggagaacctggtgattgcggtg |
|  | T7-F | Forward | aggtaatacgactcactataggg |
|  | WT-R3793 | Reverse for WT | tagtcggacacgccctgttc |
|  | M2-R3793 | Reverse for M2 | tagtccgacaccccctgttc |
